# Seta‐Inspired Mechano‐Intelligent Janus Bandage with Coordinated Adhesion–Contraction for Minimizing Scarring

**DOI:** 10.1002/adma.202505122

**Published:** 2026-05-15

**Authors:** Di Suo, Yuhe Yang, Shuai Zhao, Ho‐Pan Bei, Avery Chik‐Him Lam, Wei‐Qiang Tan, Kenneth Kak‐yuen Wong, Xin Zhao

**Affiliations:** ^1^ Department of Applied Biology and Chemical Technology The Hong Kong Polytechnic University Hong Kong SAR P. R. China; ^2^ The Hong Kong Polytechnic University Shenzhen Research Institute Shenzhen P. R. China; ^3^ Department of Plastic Surgery, Sir Run Run Shaw Hospital Zhejiang University School of Medicine Hangzhou P. R. China; ^4^ Department of Surgery, Li Ka Shing Faculty of Medicine The University of Hong Kong Hong Kong SAR P. R. China; ^5^ Research Institute For Intelligent Wearable Systems The Hong Kong Polytechnic University Hong Kong SAR P. R. China

**Keywords:** adhesion–contraction coordination, mechano‐intelligent Janus bandage, tension modulation, wound healing, wound protection

## Abstract

While advances have been made in mechano‐active and gecko‐inspired wound dressings, achieving dynamically coordinated adhesion–contraction coupling within a single‐material, stimulus‐free system with quantitatively programmable contractile output remains an unmet challenge. Here, we engineer bioinspired mechano‐intelligent Janus bandages (MIBs) with dynamically coordinated adhesion–contraction for effective wound healing. The MIBs are fabricated through micromolding of poly(lactide–co–propylene glycol–co–lactide) dimethacrylates (PmLnD), featuring an interior surface with a gecko‐mimicking wedged structure. Upon application, the MIBs recapitulate the gecko locomotion principle to achieve precise control of contractile forces with dynamically coordinated adhesion–contraction. The simply pre‐strained MIB can precisely program its intrinsic contractile force, while adhesion strength proportionally responds to the contractile force through enhanced van der Waals interactions and interfacial friction. This coordinated mechanism promotes healing in rat and porcine full‐thickness skin defect models by accelerating re‐epithelialization and enhancing angiogenesis. Mechanistically, the MIBs reduce focal adhesion kinase (FAK) expression, thereby regulating downstream pathways related to wound healing progression, including nuclear factor kappa B (NF‐κB), Wnt, and transforming growth factor‐beta (TGF‐β) pathways, enabling scar‐attenuated wound healing. We envision that this Janus design, which integrates strain‐programmable contraction with reversible gecko‐inspired adhesion, offers a useful addition to current mechanobiological strategies for wound management and soft tissue repair.

## Graphical abstract

We developed a bioinspired mechano‐intelligent Janus bandage (MIB) with a hydrophobic flat exterior and a hydrophilic interior featuring gecko‐mimicking wedged microstructures, fabricated via micromolding and anisotropic plasma treatment. The MIB dynamically synchronizes adhesion and contraction, enabling precise force modulation. It demonstrates secure yet reversible adhesion, enhanced wound closure, and angiogenesis promotion, effectively programming minimized‐scarring wound healing through mechanotransduction regulation.

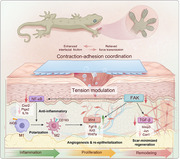

## 1 Introduction

Skin wound management remains a significant public health challenge and has a profound impact on both patients and society [1]. Clinically, the pivotal process of wound healing is wound closure, which is crucial for accelerating healing and minimizing scarring [2]. Sutures, the conventional method for wound closure, have been used for decades [3]. Despite their longstanding application, sutures exhibit several limitations, including labour‐intensive application, restricted patient mobility, and potential complications associated with uneven or uncontrolled wound tension [4]. Such irregular tension can stimulate the overproduction of pro‐inflammatory cytokines (e.g., interleukin‐1 (IL‐1)), further hindering wound healing with fibrotic scar formation [5]. Alternatively, adhesive bandages (e.g., 3M Steri‐Strip reinforced skin closure strip) have gained attention as a user‐friendly and effective solution for wound closure [6]. However, they only provide limited wound closure strength (e.g., ∼5 kPa) with partially mitigated wound tension due to passive contractile force generation, rendering them less effective, particularly for the treatment of extensive wounds with significant tension [7, 8].

To address these limitations, a growing body of research has focused on mechano‐active wound dressings capable of exerting active contractile forces to modulate wound tension [9, 10]. Recent years have witnessed significant progress in this field, including thermoresponsive tough adhesive hydrogels that contract at physiological skin temperature [10, 11, 12], hydration‐triggered shape‐memory patches that program contractile output for rapid wound closure [9, 13, 14], and enzyme‐triggered contractile hydrogel patches for diabetic wound management [15]. These studies collectively establish that mechano‐active bandages operating through mild, physiologically compatible stimuli can effectively drive wound contraction and modulate mechanosensitive pathways including the focal adhesion kinase (FAK) cascade [10, 16]. Despite these significant advances, precisely programming and tuning the contractile stress output, particularly across a specific mechanotransductive range (e.g., 30–640 Pa) relevant for fibroblast and keratinocyte responses [17, 18], within a fully stimulus‐free, pre‐strain‐based system remains an open challenge [19]. This precision is critical because excessive contractile stress can disrupt cellular homeostasis and lead to fibrosis, while insufficient stress fails to activate the necessary mechanotransduction cascades for effective wound healing. Consequently, mechano‐active bandages that enable both simple activation and precise control of contractile stress hold significant clinical potential for achieving optimal wound closure and effective regulation of wound tension.

In the application of mechano‐active bandages, adhesion plays a crucial role [20]. It not only enables secure attachment to the wound to avoid the need for complex external fixation measures (e.g., staples and medical tapes), but also serves as a vital interface between the wound and the bandage [21]. Such an interface is essential for transmitting the contractile force generated by the bandage to the wound, thereby allowing for effective regulation of wound tension. Currently, most mechano‐active bandages rely on chemical adhesives (e.g., cyanoacrylate glues and chemical bonding agents) [22, 23]; however, these approaches often complicate material composition, hinder clinical translation, and pose challenges for controlled detachment [24]. Moreover, current mechano‐active bandages often fail to intelligently provide coordinated adhesion strength in response to contraction. Achieving such coordinated adhesion would enable the effective transmission of contractile forces while avoiding either insufficient adhesion leading to compromised wound contraction, or excessive adhesion causing surrounding tissue damage [25, 26].

Recently, the remarkable ability of geckos to adhere to nearly any surface has inspired the development of gecko‐inspired bandages (GIBs) [27]. By mimicking the unique setal structures found on gecko feet, these bandages generate van der Waals forces and interfacial friction, providing strong and reversible adhesion [28]. By modulating the interaction between the setae and the surfaces, these bandages can rapidly switch between adhesion and de‐adhesion, enabling controlled attachment and removal. Moreover, gecko‐inspired biomedical adhesives have long demonstrated the translational value of microstructured adhesion for tissue‐facing applications [29, 30], and recent highly compliant adhesive patch platforms have further underscored that matching the stress profile of the adhesive interface to the dynamic compliance of living tissues is a critical determinant of performance [31, 32]. However, within the wound‐dressing field, gecko‐inspired designs have been used predominantly to improve interfacial attachment and reversible adhesion [33, 34, 35], whereas their integration with strain‐programmable wound contraction and contraction‐strengthened adhesion has been explored less extensively (Table S1). This distinction is important because wound closure performance depends not only on whether a bandage adheres, but also on how efficiently generated contractile forces are coupled to tissue and how safely the dressing can be removed after therapy.

**FIGURE 1:**
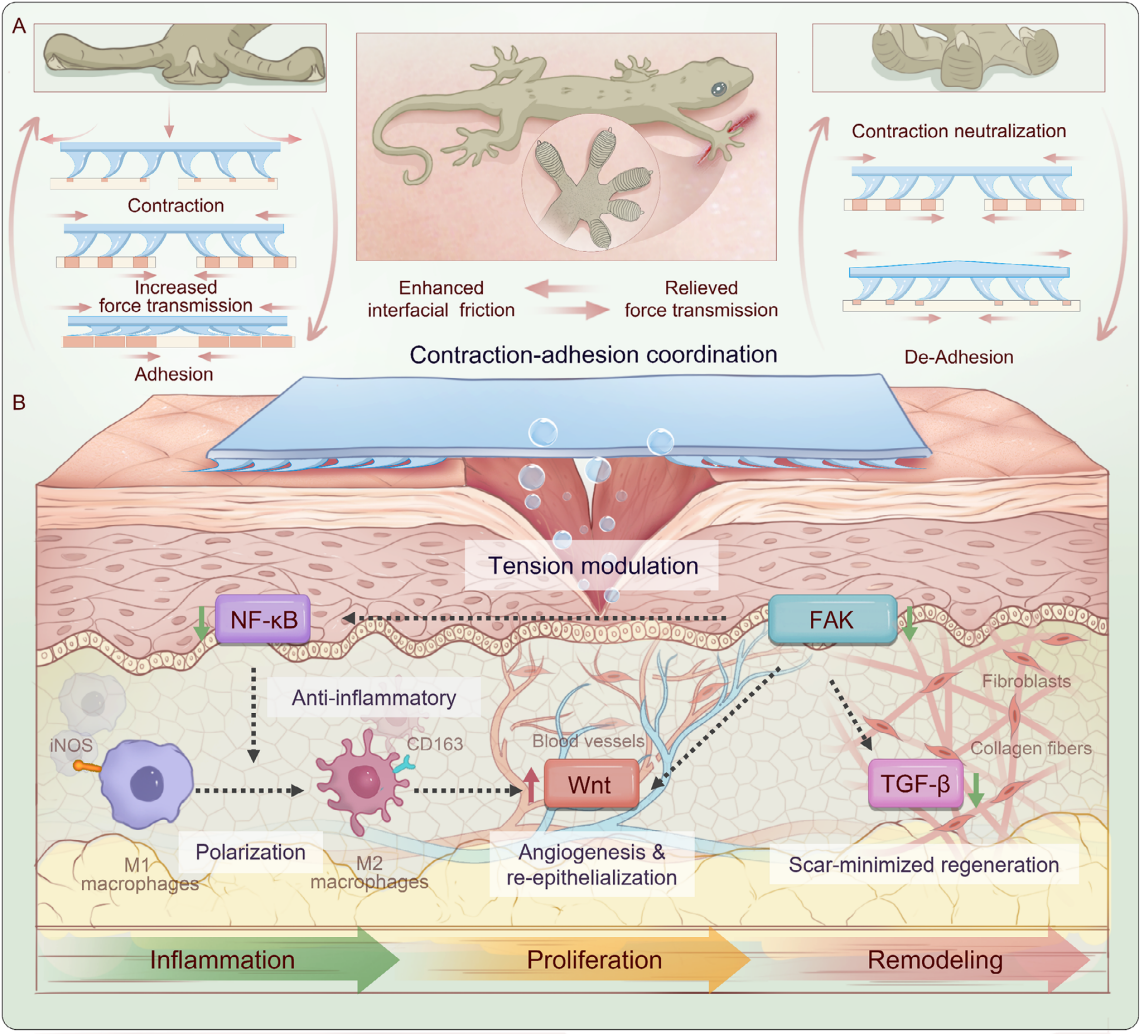
Schematic showing the mechano‐intelligent Janus bandage (MIB) for wound healing with minimized scarring via dynamically coordinated adhesion–contraction and mechanobiological regulation. (A) Bioinspired design and dynamic adhesion–contraction coordination. MIB leverages dynamic adhesion–contraction coordination by integrating a gecko‐inspired wedge microstructure for enhanced adhesion and an elastic exterior for programmable contraction. Upon application, stretch‐triggered contraction mimics the gecko locomotion and synchronizes adhesion and wound tension relief, while reversible detachment is achieved by counteracting contractile forces through controlled post‐strain. (B) Mechanobiological regulation of scar‐minimized healing. MIB modulates mechanobiological signaling across wound healing phases, promoting macrophage polarization to reduce inflammation, enhancing angiogenesis and re‐epithelialization during proliferation, and regulating matrix remodeling to prevent fibrosis. This coordinated adhesion–contraction mechanism optimally balances mechanical forces and biochemical cues, facilitating efficient tissue repair.

Here, we engineer a bioinspired mechano‐intelligent Janus bandage (MIB) with dynamically coordinated adhesion–contraction to minimize scarring while enhancing wound healing (Figure 1). The MIB is fabricated from fluid, photocrosslinkable poly (lactide–co–propylene glycol–co–lactide) dimethacrylates (PmLnD) by micromolding. PmLnD is used due to its excellent biocompatibility and high elasticity. The MIB features an interior surface with a gecko‐inspired bidirectional wedge array (50 µm for height and width) to apply tissue adhesion and a flat external surface for stretchability. Upon stretching and application, the MIB recapitulates the gecko locomotion principle to strategically harness the centripetal contractile force of the bandage to maximize the interfacial forces during contraction to achieve coordinated adhesion–contraction (Figure 1A). The elastic MIB can precisely program the intrinsic wound closure strength from 0 to 89 kPa by simple pre‐strain (from 0% to 35%). The adhesion strength is found to proportionally respond to the contractile force due to the strengthened van der Waals interactions and interfacial friction between the wedged structures and the tissue. This dynamic mechanism enables robust wound closure while effectively modulating wound tension. Additionally, the MIBs can be removed without damaging the wound tissue by reverse post‐strain to counteract the contractile force to relieve adhesion strength. Our MIBs demonstrate substantial potential to promote effective healing in both rat and porcine full‐thickness skin defect models by regulating wound tension with inhibition of mechanosensitive FAK expression, which comprehensively modulates the wound healing progression (Figure 1B). Specifically, during the inflammatory phase, downregulated FAK suppresses the downstream inflammatory NF‐κB pathway, driving resting macrophages towards the M2 phenotype and facilitating the transition of the wound healing cycle from the inflammatory phase to the proliferative phase. Subsequently, in the proliferative phase, decreased FAK expression upregulates the Wnt pathway, significantly enhancing angiogenesis and re‐epithelialization for accelerated wound healing. Finally, during the remodelling phase, reduced FAK expression downregulates the TGF‐β pathway, which is critical for achieving balanced matrix degradation and deposition, eventually inhibiting scar formation.

By unifying gecko locomotion principles with pre‐strain‐based mechanical programming in a single micromolded elastomeric platform, this work introduces a Janus bandage design in which strain‐programmable contraction and reversible adhesion are not merely co‐present but dynamically coordinated through the proportional coupling mechanism. This mechanobiological strategy opens new avenues for skin wound management and holds broad potential for the treatment of wounds in other soft tissues.

## 2 Results and Discussion

### 2.1 Design, Fabrication and Biocompatibility Evaluation of MIBs

To fabricate the bioinspired mechano‐intelligent Janus bandage (MIB), we used photocrosslinkable PmLnD developed by our group, where m and n refer to the unit length of propylene glycol and the molar ratio of lactide to polypropylene glycol (PPG), respectively [36, 37]. Four PmLnD formulations with varying m/n ratios (7/2, 17/4, 34/4, and 68/8) were synthesized and their structures were confirmed through Fourier‐transform infrared (FTIR) and proton nuclear magnetic resonance (^1^H NMR) spectroscopy (Figure S1A,B). To enable quantitative structural verification, ^1^H NMR peak integration was performed to determine both the PPG/LA segment composition and the methacrylate functionalization efficiency (Figure S1B and Table S2). The PPG backbone resonance at ∼3.56 ppm (3H per PPG repeat unit) and the LA methyl resonance at ∼1.58 ppm (6H per Ln) were integrated to determine m = ∫_3.56_/3 and n = ∫_1.58_/6, which matched the targeted formulations. Methacrylation was quantified by integrating the vinyl protons at ∼6.21 and ∼5.60 ppm, giving high methacrylation efficiencies of 96.75–99.50% (Table S2). FTIR collected during photocuring (0, 60, and 120 s) showed a time‐dependent attenuation of the vinyl‐associated band at ∼1640 cm^−1^, confirming efficient consumption of methacrylate C═C groups and crosslinked network formation (Figure S2). The viscosity of each PmLnD formulation was measured to assess their flowability, with all samples exhibiting values below 800 mPa·s. This level of viscosity enabled precise micromolding into various shapes (Figure S1C,D). Tensile testing was subsequently performed on the formulations, revealing that P68L8D possessed the greatest elasticity, as evidenced by the highest elongation‐at‐break (∼145%) and a moderate tensile modulus (∼0.8 MPa), which is comparable to that of commercial elastic bandages (Figure S1E–G) [38]. Therefore, we selected P68L8D as the base material for our MIB and denoted it as PLD for simplicity in the following studies.

**FIGURE 2:**
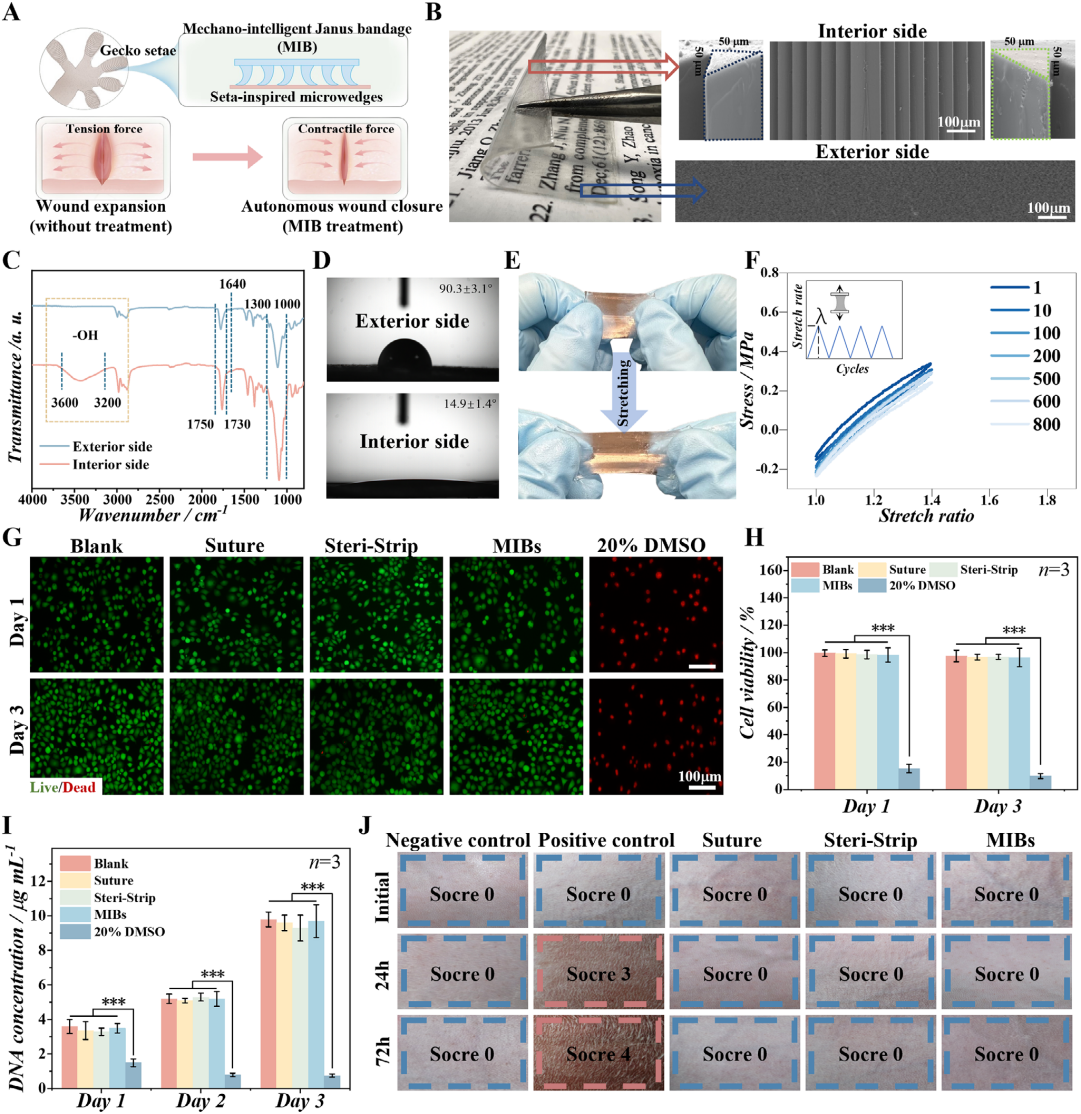
Design, fabrication, and biocompatibility evaluation of the contractile MIBs. (A) Bioinspired design of the MIBs. (B) The macroscopic and SEM images showcase the structure of the fabricated MIBs. (C) FTIR and (D) water contact angles of the different surfaces (the gecko‐inspired microstructured side and the flat side) of the optimized MIB. (E) Tensile property demonstration and (F) cyclic tensile evaluation of both surfaces (the gecko‐inspired microstructured side and the flat side) of the optimized MIB. (G) Live/Dead staining of the L929 fibroblasts showing cytocompatibility of MIBs. Green and red fluorescence indicated the live and dead cells, respectively. Quantification of (H) cell viability and (I) cell proliferation in 1–3 days. (J) Photographic evidence presenting skin irritation outcomes of various materials applied to the dorsal skin of New Zealand rabbits. All experiments were conducted with a sample size of *n* = 3 and analyzed using two‐way ANOVA followed by Tukey's post hoc test for multiple comparisons. Data are expressed as mean ± SD, with statistical significance denoted as ***p < 0.001.

Next, the MIB was fabricated using a micromolding technique, employing a template with gecko‐inspired wedges produced by precision mechanical machining (Figure S3A). Notably, the wedges were uniquely designed with a bidirectional configuration to strategically harness the centripetal contractile force of the bandage to maximize van der Waals interactions and interfacial friction during contraction to achieve coordinated adhesion–contraction (Figure 2A). The Janus bandages were then fabricated through two‐step micromolding with PDMS and PLD (Figure S3B). Subsequently, we applied plasma treatment to the interior side of the PLD patch with gecko wedges, rendering it hydrophilic to enhance the interaction between the wedged structure and skin tissue (Figure S3C) [39]. In contrast, the exterior side retained its hydrophobic nature, resulting in the MIBs with distinct structural and chemical properties on each side.

The surface structures of the MIB were characterized by scanning electron microscopy (SEM) (Figure 2B). The interior surface presented a precise bidirectional wedge structure, with each wedge having a height and width of 50 µm, effectively mimicking the adhesive mechanism of gecko setae for tissue adhesion. In contrast, the exterior side exhibited a smooth and dense structure, providing a beneficial barrier for wound protection. The chemical structures of both sides were analyzed using Fourier‐transform infrared (FTIR) spectroscopy (Figure 2C). After plasma treatment, the interior side of the MIB showed a robust characteristic ‐OH peak at 3600–3200 cm^−^^1^, indicating significant hydrophilicity with a contact angle of less than 15° (Figure 2D). Given that plasma‐induced hydrophilicity may undergo hydrophobic recovery, we evaluated the wettability stability of both the interior and exterior surfaces of the MIBs after vacuum‐drying and hermetic storage. The plasma‐treated interior surface maintained a low water contact angle after 20 days, changing only slightly from 15.8° (day 0) to 17.3° (day 20), whereas the exterior surface remained hydrophobic, confirming durable surface anisotropy over the tested period (Figure S4). To further support the intended function of the hydrophilized micro‐wedged interface under exudative conditions, we evaluated the dynamic wetting behavior by time‐lapse contact‐angle measurements. The untreated surface maintained a high contact angle (∼90°), whereas the plasma‐treated tissue‐contacting surface exhibited a pronounced reduction in contact angle over time, indicating rapid wetting/spreading (Figure S5). This fast‐wetting behavior is consistent with efficient water guidance along the hydrophilized microgrooves, which facilitates displacement and drainage of interfacial exudate and thereby promotes intimate tissue contact during wet‐surface attachment (Video S1). Stretchability is a critical property for elastic wound bandages, as it enables the bandage to conform to the body's movements and maintain effective coverage over joints and other flexible areas (Figure 2E) [40]. Our MIB exhibited exceptional stretchability and elasticity, withstanding over 140% strain (Figure 2F). Furthermore, it demonstrated robust cyclic stretching performance, having endured more than 800 cycles, ensuring long‐term durability throughout the healing process. More importantly, this outstanding cyclic elasticity enables the precise programming of contraction forces through pre‐straining the wound bandages prior to application.

To highlight the advantages of the selected morphology, two additional bandage types with distinct surface structures were fabricated: a Flat‐Flat bandage, featuring flat surfaces on both sides (i.e., pure P68L8D), and a Microgroove‐Flat bandage, incorporating biomimetic microgrooves on one side and a flat surface on the other (Figure S6A). SEM analysis verified that all bandage types exhibited well‐defined and precise surface morphologies, underscoring the excellent micromolding properties of P68L8D (Figure S6B). In addition, tensile property analysis revealed that the strain values and elastic modulus of the pure component P68L8D (i.e., Flat‐Flat bandages) were similar to those of the MIB and Microgroove‐Flat bandages (Figure S6C). This observation is expected, as surface microstructures do not significantly influence the bulk mechanical properties of the bandage material. However, despite these similar mechanical properties, the MIB exhibited significantly superior adhesion performance compared to both the Flat‐Flat and Microgroove‐Flat bandages (Figure S6D). This enhancement is attributed to the increased van der Waals interactions and interfacial friction between the gecko‐inspired wedged microstructures and the tissue surface. Consistent with these quantitative results, direct adhesion tests on finger joints further visually confirmed this advantage. As shown in Figure S6E, only the MIB maintained stable adhesion on the curved finger joint, whereas the Flat‐Flat and Microgroove‐Flat bandages failed to adhere. This finding underscores the key advantage of the wedged microstructures in ensuring reliable attachment to dynamic and irregular surfaces. Collectively, these results highlight the critical importance of the MIB's unique morphology in promoting effective wound healing.

In addition, biocompatibility is the paramount consideration for wound bandages to avoid any adverse reaction when in contact with the skin and surrounding tissues [41]. Thus, to systematically assess biocompatibility in accordance with the ISO 10993 standard, in vitro evaluations were conducted using L929 fibroblasts [42] (Figure 2G). Compared to the control groups (i.e., suture and 3M Steri‐Strip), the MIB group demonstrated over 95% cell viability and a similar proliferation rate from day 1 to day 3, as confirmed by Live/Dead staining and PicoGreen evaluation (Figure 2H,I). Biocompatibility was further evaluated using a rabbit skin irritation model (Figure 2J) [42]. No signs of inflammation were observed at 24 and 72 h after MIB application, indicating that our MIB was skin‐friendly without any irritation. Collectively, these results suggested that our MIB exhibited excellent biocompatibility, making it suitable for application to wound sites. In summary, we successfully fabricated the MIB with anisotropic gecko‐inspired structures, excellent elasticity, and outstanding biocompatibility.

### 2.2 Maintenance of a Sterile and Moist Environment With MIBs

Current mechano‐active wound bandages often face challenges such as biofouling, bacterial invasion, and uncontrollable dehydration after application, which can delay the healing process and increase the risk of scar formation [19, 43]. We hypothesized that our MIBs could function as an epidermal analog to effectively preserve an ideal moist and sterile environment, which is a part of the orderly progression in the wound healing cascade (Figure S7A). Thus, antifouling performance of the MIB was initially assessed by measuring protein adsorption with fluorescein isothiocyanate‐labeled bovine serum albumin (FITC‐BSA) as a model foulant [44]. Compared to the commercial Steri‐Strip, which exhibited pronounced fluorescence signals indicative of substantial protein adsorption, the MIB group with its dense and hydrophobic exterior surface showed negligible fluorescence, demonstrating the MIB's excellent antifouling capacity (Figure S7B,C). To evaluate the efficacy of the MIB in preventing bacterial invasion, an in vitro antibacterial adhesion assay was performed by culturing Gram‐negative *E. coli* and Gram‐positive *S. aureus* on the surfaces of various sample groups [45]. After 48 h of incubation, the MIB demonstrated minimal adhesion of both bacterial species, a result attributed to the hydrophobic characteristics of PLD (Figure S7D,E). Moreover, wound bandages should maintain a moist environment after application while retaining their structural integrity and functionality, which is crucial for effective wound healing [46]. The moisture retention capacity of the MIBs was evaluated by measuring their water vapor transmission rate (WVTR) (Figure S7F). The suture and the Steri‐Strip groups exhibited uncontrollable WVTRs (over 130 g/h/m^2^), which could lead to rapid wound dehydration. In contrast, MIBs demonstrated a moderate WVTR of ∼87 g/h/m^2^, attributed to their unique Janus structure and hydrophobicity of PLD. This value falls within the optimal range for wound dressings (∼74–95 g/h/m^2^), indicating that MIBs effectively maintain appropriate humidity levels to support wound healing [47]. Finally, swelling resistance of the MIB was assessed under moist conditions. In contrast to the commercial Steri‐Strip, the MIB maintained its original shape after 14 days (Figure S7G), and its mechanical properties remained largely unchanged following prolonged exposure to moisture (Figure S7H). Such superior moisture retention and swelling resistance of our MIB can be attributed to its dense and hydrophobic nature, which is crucial for maintaining prolonged wound coverage and hydration.

In all, these results collectively demonstrate that our MIB exhibited excellent wound protection capabilities, including inhibited biofouling and bacterial invasion, as well as regulated moisture retention. These features are essential for the effectiveness of wound bandages for enhanced wound management.

### 2.3 Intelligent Adhesion–Contraction Coordination of MIBs

Physiologically, wound tension varies depending on the size and location of the wound, ranging from 10 to 60 kPa [48]. Therefore, for mechano‐active bandages, it is crucial to dynamically and precisely control the contractile forces according to the needs of the wound bed to achieve better wound closure and tension mitigation. Insufficient contractile forces may result in incomplete wound closure, while excessive contractile forces could cause tissue ischemia, excessive inflammation or even damage to the surrounding tissue. Simultaneously, beyond achieving precise control of contractile forces, it is equally important to ensure dynamically coordinated adhesion strength in response to contraction. Such coordinated adhesion enables effective transmission of contractile forces to prevent insufficient adhesion, which could lead to compromised wound contraction, or excessive adhesion, which could cause damage to surrounding tissues.

**FIGURE 3:**
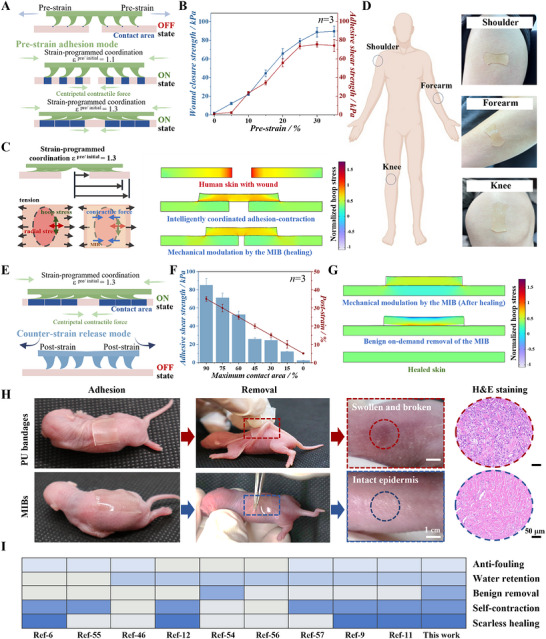
Intelligent adhesion–contraction coordination and benign removal of MIBs. (A) Illustration of synchronized adhesion and contractile forces through pre‐strain. (B) Mechanical assessment of adhesion strength and wound closure strength with varying pre‐strain. (C) Von mises stress profiles for the sealed defects upon pre‐strained MIB loading and the lateral cross‐section diagram. (D) Performance evaluation of MIBs on various body areas, confirming effective adhesion and contraction. (E) Schematic showing post‐strain reducing contact area and adhesion forces for gentle detachment. (F) Analysis of adhesion force reduction with decreasing contact area. (G) FEM modelling showing redistribution of contact area and force balance during removal. (H) Infant rat model validating minimal skin damage with MIBs compared to commercial PU bandages. MIBs could be gently removed without causing significant injury to the delicate skin of infant rats, as evaluated by careful observation and H&E staining. (I) The unique advantages of this work compared to other publications (anti‐fouling, water retention, benign removal, self‐contraction, and wound healing). The blue color represents the work with this characteristic [6, 9, 11, 12, 46, 54, 55, 56, 57]. All experiments were conducted with a sample size of *n* = 3.

In our study, we leveraged the gecko locomotion principle to achieve precise control of the contractile forces with intelligently coordinated adhesion–contraction. Specifically, during application, the MIBs were pre‐strained to generate a precise centripetal contractile force within the elastic bandage, enabled by their outstanding cyclic stretchability and elasticity (Figure S8A). Interestingly, due to our uniquely designed bidirectional micro‐wedges, the direction of the contractile force on each side of the MIB aligns with the inclination of the micro‐wedges. Such a design could maximize van der Waals interactions and interfacial friction during contraction to enhance the adhesion strength, simulating the “active mode” of gecko locomotion (Figure 3A) [49]. As the pre‐strain increases, the centripetal contractile force proportionally increases, enhancing adhesion strength for efficient force transmission to realize a coordinated adhesion–contraction mechanism. The coordinated wound closure and adhesion strengths of pre‐strained MIBs were evaluated (Figure 3B). Increasing the pre‐strain (ε) from 0% to 35% resulted in a proportional enhancement of wound closure strength, reaching up to 89 kPa. These results demonstrate that the contractile force can be precisely regulated by adjusting the pre‐strain. Furthermore, the range of the closure strength effectively covers the highly variable wound tension across different wound sizes and locations (i.e., 10 to 60 kPa) [48], which is critical for achieving precise wound closure under diverse conditions. At the same time, the adhesion strength exhibited a synergistic increase from 1 to 76 kPa. To validate the unique morphology‐driven coordination between adhesion and contraction in the MIB, the relationship between these properties was compared with those of the previously fabricated Flat‐Flat and Microgroove‐Flat bandages (Figure S9). Unlike the MIB, which exhibited proportional increases in both adhesion and contraction with increasing pre‐strain (0% to 30%), the adhesion strength of the Flat‐Flat and Microgroove‐Flat bandages did not increase accordingly and even showed a slight decrease. This reduction was likely due to enhanced contractile forces at higher pre‐strain levels, which resulted in interfacial slippage and diminished adhesion strength. In contrast, the MIB demonstrated a clear and pronounced coordination between contraction and adhesion. Such coordination is essential for the effective transmission of contractile forces, thereby ensuring optimal wound closure.

To further evaluate the applicability of MIBs for wounds of different sizes, we conducted in vitro wound closure tests with varying wound sizes (0.1 cm × 1.5 cm, 0.25 cm × 1.5 cm, and 0.5 cm × 1.5 cm) simulating different‐sized wounds commonly encountered, including those resulting from minimally invasive surgery. The results confirmed that MIBs could achieve robust adhesion and wound closure even for wounds as small as 0.1 cm × 1.5 cm, as long as the MIBs were appropriately pre‐strained (Figure S10). These findings indicated that our MIBs are effective for different‐sized wounds, broadening their potential clinical utility.

For mechano‐active bandages, it is important to regulate wound tension, as it plays a critical role in activating downstream mechanosensitive pathways (e.g., FAK). The effects of MIBs on wound tension modulation were modeled and analyzed using the finite‐element method (FEM) (Figure 3C). Notably, the frictional contact at the interface between the MIB's wedge microstructures and the wound tissue is a key determinant of adhesion, as validated by previous studies [50, 51]. The frictional force is directly proportional to the contractile force imparted by pre‐strain [52]. By precisely controlling pre‐strain, our system modulated frictional contact at the interface, enabling switchable and robust adhesion in the “activated” state. After wound formation, computational analysis of the hyperelastic solid wound model revealed a critical circumferential stress concentration exceeding 200% at the wound periphery, primarily attributed to the intrinsic pre‐tension of cutaneous tissue [9]. This phenomenon could substantially compromise early wound closure for delayed tissue regeneration. The implementation of MIBs efficiently transduced contractile forces across the tissue matrix, resulting in a homogenized stress distribution. This intervention achieved a 75.6% reduction in peak circumferential tension gradients at the wound edge, thereby creating a biomechanical microenvironment conducive to regenerative healing. FEM simulations further elucidated that a precisely controlled level of pre‐strain could ensure a critical equilibrium between the integrity of interfacial adhesion and modulation of contractile forces, effectively maintaining tissue‐level stress within physiological tolerance. In all, the FEM could faithfully capture the synchronized adhesion and contraction that underpin the bandage's wound closure efficacy. To gain insight into real‐world application, the performance of MIBs was assessed on different regions of human skin (Figure 3D). The MIB adhered effectively to the forearm, shoulder, and knees, maintaining strong adhesion despite varying local tension. Upon release, the MIB contracted instantly, providing robust compression and demonstrating its capacity to generate sufficient force for effective wound closure.

In addition to coordinated adhesion–contraction during adhesion, it is also crucial to ensure benign removal to minimize tissue damage and patient discomfort [53]. To further understand its usability, we investigated the de‐adhesion behavior of the MIB during removal (Figure S8B). Our MIB demonstrated reconfiguration of the micro‐wedge array by inverse post‐strain, effectively nullifying centripetal contractile forces at the biointerface (Figure 3E). This phase transition comprised a cascade of interfacial energy dissipation: (1) exponential decay of van der Waals interactions, (2) attenuation of frictional forces, and (3) reduction in adhesive strength, collectively enabling gecko‐inspired topological disengagement with ultralow delamination energy [49, 51]. We further quantified the effect of post‐strain on the detachment force and observed that with increasing post‐strain (decreasing contact area), the required detachment force gradually decreased (Figure 3F). The FEM analysis demonstrated that post‐strain could trigger a mechano‐geometric phase transition, leading to the redistribution of interfacial stresses at the interface (Figure 3G). Specifically, upon the removal of the MIB, the release of the pre‐applied contractile force resulted in a proportional reduction of frictional force at the interface, thereby decreasing the overall adhesion force. This process initiated a rapid redistribution of interfacial stress, transitioning from a concentrated stress state during adhesion to a more relaxed state after removal. Interestingly, even though only a slight elongation of the MIB was observed during the removal process, it was sufficient to cause a substantial decrease in the contractile force at the interface. This phenomenon was reflected in stress profiles, where localized interfacial stresses dissipated rapidly once the constraint was released. The dynamic change in stress distribution highlighted the smart and facile control over the removal process of our MIBs.

To further validate the benign removal enabled by our MIB, detachment analysis using an infant rat model was adopted (Figure 3H). This model was used here specifically as a sensitive, delicate‐skin model for evaluating atraumatic detachment [54]. All procedures were performed under anesthesia to minimize animal discomfort. Analysis of the infant rat skin condition and hematoxylin and eosin (H&E) staining revealed that the MIB could be gently removed without causing significant damage to the delicate skin, whereas commercial bandages exhibited irreversible adhesion that resulted in notable skin injury [54]. In addition, reverse post‐strain detachment was demonstrated on hair‐bearing human skin, showing stable adhesion and gentle, controlled removal without visible skin irritation or hair removal (Video S2). Such benign on‐demand removal of the MIBs can be potentially beneficial in the care of chronic wounds in clinical settings where frequent wound dressing changes are required.

In summary, these results demonstrate that our MIB can intelligently recapitulate the gecko locomotion mechanism to precisely program the adhesion–contraction coordination, which is crucial for the successful implementation of the bandage. Finally, a comprehensive comparison with other mechano‐active elastic bandages (Figure 3I) demonstrated that the MIB excels in key areas such as anti‐fouling, water retention, gentle removal, self‐contraction, and wound healing. These synergistic properties facilitate effective wound closure and promote wound healing, underscoring the MIB's substantial translational potential.

### 2.4 Enhanced Scar‐Attenuated Regeneration in Full‐Thickness Rat Skin Wound Model

**FIGURE 4:**
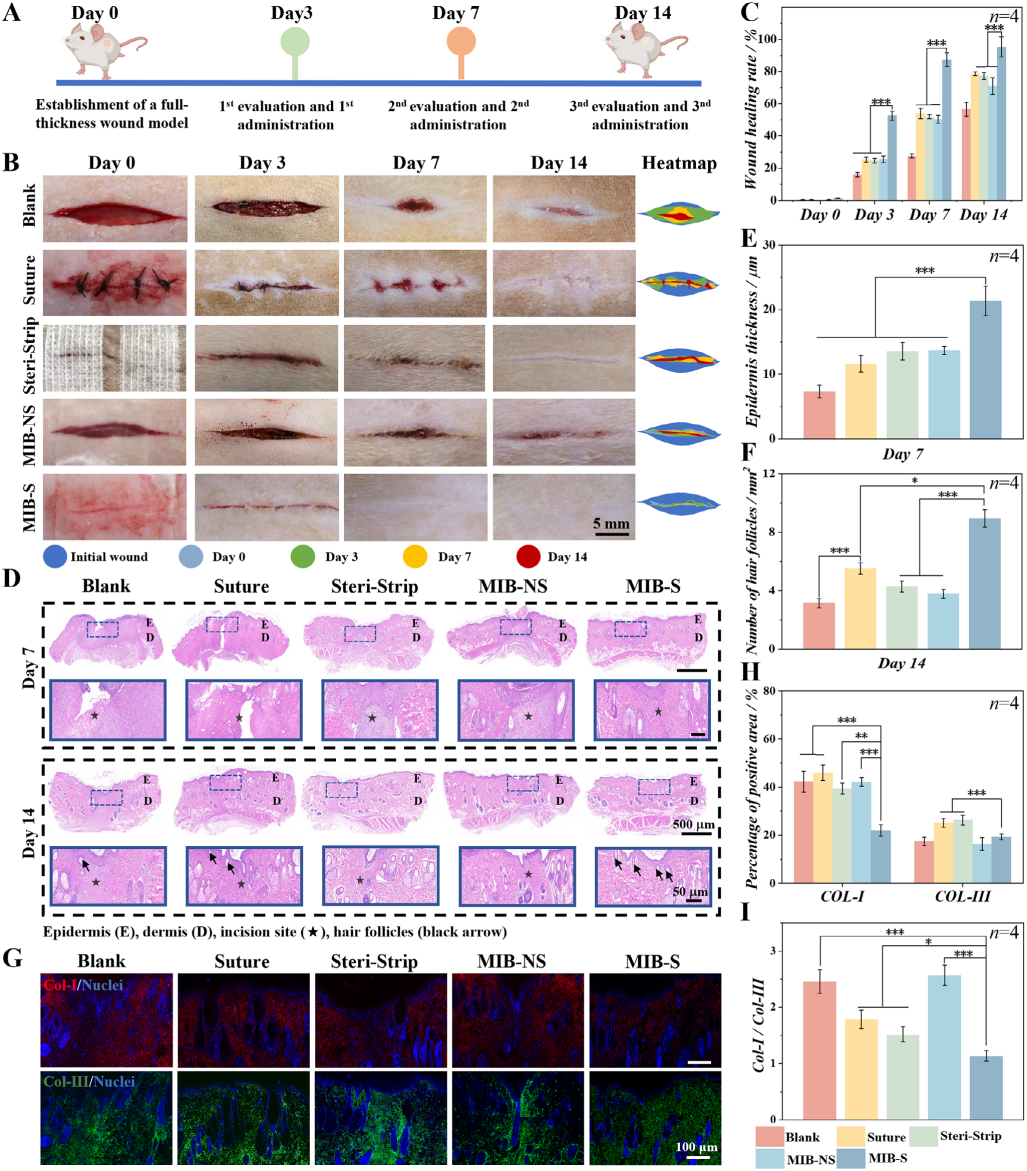
Therapeutic efficacy of MIBs on scar‐attenuated wound healing in a full‐thickness rat skin wound model. (A) Schematic illustration showing the construction of full‐thickness skin defect SD rat model. (B) Representative wound images and the wound healing map treated with different samples. (C) Quantification of the wound healing rate of different samples calculated by the wound area reduction percentage. (D) H&E staining of wound sections after 7‐ and 14‐day of treatment. The second row shows the magnified image of the marked area. The stars indicate incision area, and the black arrows indicate the hair follicles. Quantification of (E) epidermis thickness and (F) number of hair follicles in H&E staining. Immunofluorescence staining of (G) Col‐I and Col‐III for the wound sections after 14 days. (H) Quantification of the positive area percentage, performed by calculating the proportion of the tissue section exhibiting specific immunofluorescence signal relative to the total area of the tissue section analyzed. (I) Ratio between the Col‐I and Col‐III. All experiments were conducted with a sample size of *n* = 4 and analyzed using a one‐way or two‐way ANOVA followed by Tukey's post hoc test for multiple comparisons. Data are expressed as mean ± SD, with statistical significance denoted as *p < 0.05, **p < 0.01, and ***p < 0.001.

The previous results demonstrated that our MIBs exhibited excellent performance in robust wound closure with tension modulation. To further evaluate therapeutic efficacy, a full‐thickness rat skin wound model was selected, as it enables convenient and well‐established assessment of wound closure dynamics, histological evaluation of healing quality, hair follicle regeneration, and facilitates mechanistic studies. After establishing the model, sutures and commercial Steri‐Strip bandages were applied as controls. The experimental group employed the MIB with a 20% pre‐strain (designated as MIB‐S), which could generate contractile forces to effectively facilitate the precise approximation of wound edges, ensuring complete closure. A non‐strain MIB group (designated as MIB‐NS) served as a control to assess the role of contractile forces in wound healing. The wound healing process was systematically evaluated on days 3, 7, and 14 (Figure 4A). Macroscopic images revealed that the MIB‐S group demonstrated remarkable wound closure as early as day 3, with uniform and continuous healing observed throughout the experiment (Figure 4B). By day 14, complete wound closure was achieved in this group, indicating that the MIB‐S more effectively promoted tissue repair and re‐epithelialization compared to its counterparts. In contrast, the MIB‐NS groups exhibited substantial non‐healing areas with evident discontinuities. Furthermore, the quantified results of the healing rate confirmed that the MIB‐S group significantly accelerated the wound healing process (Figure 4C).

Histological staining was performed to assess wound healing progression. On day 7, hematoxylin and eosin (H&E) staining revealed that the MIB‐S group exhibited well‐defined granulation tissue regeneration and the smallest gap between dermal layers among all groups (Figure 4D). By day 14, the MIB‐S group displayed more structured epidermal tissue and significantly enhanced hair follicle regeneration. Quantitative analysis revealed that the MIB‐S group achieved the greatest epidermal thickness (∼22 µm), 1.6 times greater than that of the MIB‐NS group, and closely approximating the thickness of healthy skin (∼25 µm) (Figure 4E) [58]. Additionally, the MIB‐S group demonstrated significantly higher hair follicle density (∼ 8.9/mm^2^), which was approximately 2.1‐fold and 2.4‐fold greater than that in the Steri‐Strip and MIB‐NS groups, respectively (Figure 4F). Since hair follicle regeneration is a key indicator of skin appendage restoration and functional healing, the hair follicle density in the MIB‐S group approached that of normal skin (∼9.7/mm^2^), suggesting improved skin integrity and regenerative potential [59]. Further analysis of collagen organization, a key component of skin, was conducted using Masson's trichrome staining. The MIB‐S group exhibited significantly greater collagen deposition and more uniform fibrous tissue formation, indicating enhanced extracellular matrix assembly, while the MIB‐NS group and other groups demonstrated markedly reduced collagen deposition and irregular fibrous tissue organization (Figure S11A,B). Additionally, restoring the mechanical properties of the healed tissue is another key consideration in wound repair strategies [60]. The mechanical strength of the newly formed tissue was evaluated using an incision breaking strength test (Figure S11C). The MIB‐S group exhibited the highest breaking strength (∼7.3 kPa), surpassing Steri‐Strip and MIB‐NS groups by 1.4‐fold and 1.9‐fold, respectively. This result highlighted the enhanced mechanical integrity of the regenerated tissue, closely approximating that of normal skin. To further elucidate the scar‐attenuated extracellular matrix remodeling, we performed immunofluorescence staining for collagen I (Col‐I) and collagen III (Col‐III) (Figure 4G). The MIB‐S group exhibited significantly reduced Col‐I expression and enhanced Col‐III synthesis compared to other groups, achieving a Col‐I/Col‐III ratio (∼1.1) closely resembling that of normal skin (∼1.2) (Figure 4H,I) [61].

Overall, these results underscore the ability of MIB‐S, through the exertion of critical contractile forces, to accelerate the wound healing process by promoting granulation tissue regeneration, regulating collagen deposition, and facilitating balanced matrix remodeling. This finally supports functional tissue regeneration while reducing fibrotic scarring.

### 2.5 Bioinformatic Analysis of the Scar‐Attenuated Wound Healing Mechanisms

**FIGURE 5:**
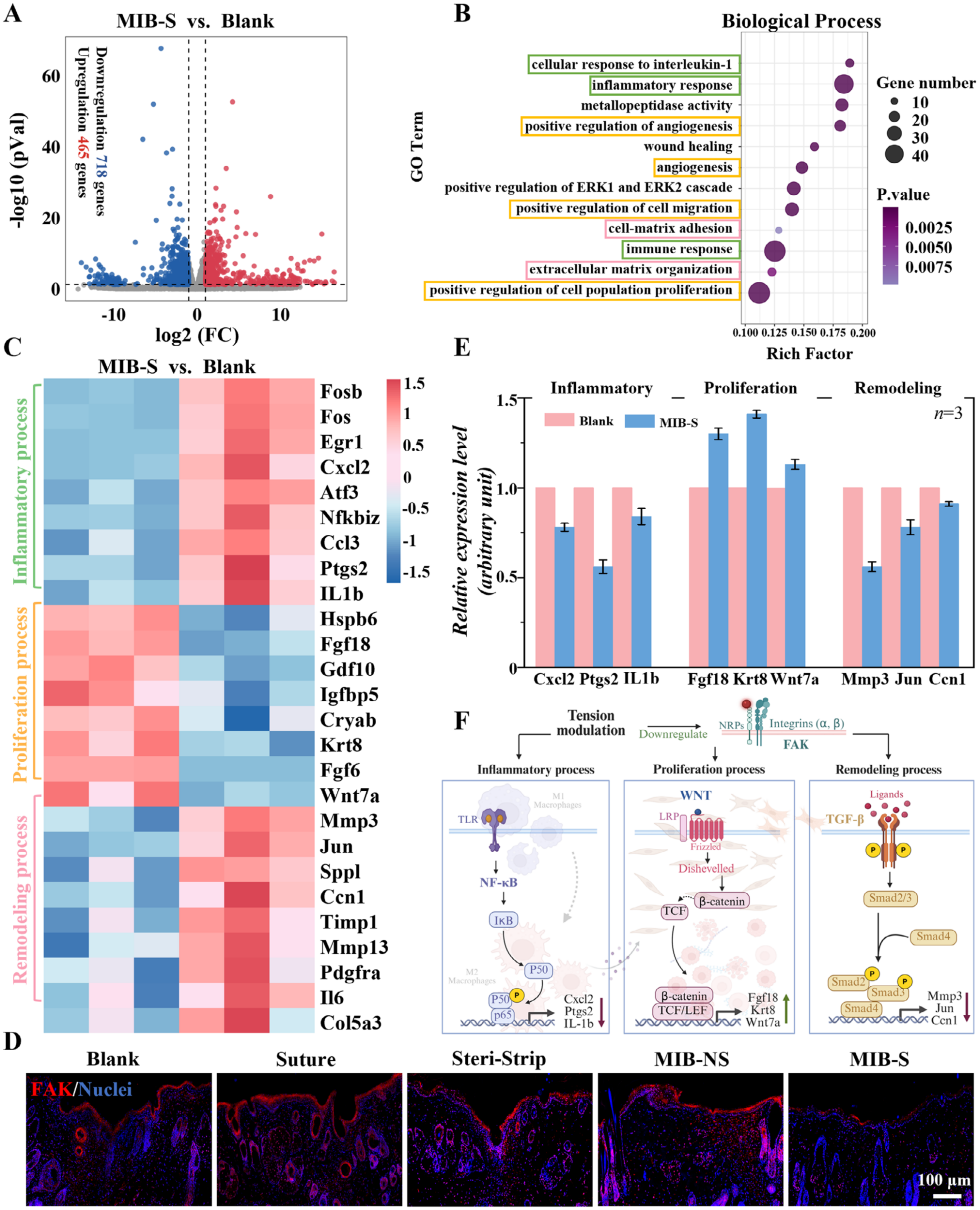
Transcriptomic and mechanistic analysis of the MIBs in promoting scar attenuated wound healing. (A) Volcano plot highlighting differential gene expressions (DGEs) between MIB‐S and blank control on day 7. (B) GO enrichment analysis showing biological processes involved in wound healing. (C) Heatmap depicting gene expression changes associated with the inflammatory, proliferative, and remodeling phases of wound healing progression. (D) Immunofluorescence FAK staining for the wound after 7 days. The red and blue colors indicate the FAK and nuclei, respectively. (E) Quantitative expression levels of representative genes from the inflammatory (e.g., *Cxcl2*, *Ptgs2*, *IL*‐*1b*), proliferative (e.g., *Fgf18*, *Krt8*, *Wnt7a*), and remodeling (e.g., *Mmp3*, *Jun*, *Ccn1*) phases. (F) Schematic illustrating MIBs with tension modulation for reduced FAK expression to further regulate wound healing‐related pathways such as the NF‐κB, Wnt, and TGF‐β pathways for scar attenuated wound regeneration. All experiments were conducted with a sample size of *n* = 3.

To investigate the underlying mechanisms of MIB‐mediated scar‐attenuated wound healing, bulk RNA‐seq analysis was performed. Comparison of the MIB‐S group with the blank group revealed 465 upregulated and 718 downregulated differentially expressed genes (DEGs) (Figure 5A). GO analysis revealed several differentially expressed processes related to wound healing progression such as inflammation (e.g., inflammatory response and immune response), proliferation (e.g., angiogenesis and positive regulation of cell migration), and remodeling (e.g., cell‐matrix adhesion and extracellular matrix organization), indicating the critical role of MIB in wound healing regulation (Figure 5B) [11]. Moreover, Kyoto Encyclopedia of Genes and Genomes (KEGG) enrichment analysis implicated several pathways related to immunoregulation (e.g., nuclear factor kappa B (NF‐κB)) and mechanotransduction (e.g., focal adhesion signaling pathway (FAK)) (Figure S12).

To gain deeper insight into the modulation process of MIBs on wound healing, the DEGs within these pathways were clustered in a heatmap and categorized into three biological processes: inflammatory, proliferative, and remodeling (Figure 5C). Specifically, in the inflammatory phase, downregulation of several pro‐inflammatory genes (e.g., *Cxcl2*, *Ptgs2*, and *IL*‐*1b*) was observed, indicating that clear inflammation suppression was induced by MIBs. This could be attributed to the downregulation of the pro‐inflammatory NF‐κB pathway as depicted in the KEGG analysis. Previous studies have reported that excessive tension during wound healing can over‐activate FAK expression, subsequently triggering downstream inflammatory cascades (e.g., NF‐κB pathway) [62, 63]. Given that MIBs can mitigate wound tension, they may downregulate FAK expression, thereby suppressing NF‐κB signaling and reducing inflammation. Thus, the expression of FAK in different groups was first validated by immunofluorescence staining. As expected, significantly inhibited FAK expression was observed in the MIB‐S group, which may further contribute to regulation of the NF‐κB pathway and inhibition of inflammation (Figure 5D; Figure S13). The reduced inflammation is critical for wound healing progression. Immunofluorescence staining for CD68 (M0 marker), iNOS (M1 marker), and CD163 (M2 marker) was also performed, revealing that M2 macrophage polarization was significantly enhanced by MIBs, with a reduced M1 ratio (9.6 ± 1.12%) and an elevated M2 ratio (17.6 ± 1.32%) (Figure S14). This shift toward M2 dominance could promote the transition from the inflammatory phase to the proliferative phase, thereby accelerating wound healing.

Subsequently, during the proliferation process, we identified significant upregulation of genes such as *Fgf18*, *Krt8*, and *Wnt7a*, which are associated with promoting vascular network formation and re‐epithelialization (Figure 5E). Notably, *Wnt7a*, a key ligand of the Wnt pathway, can be released by polarized M2 macrophages to enhance tissue regeneration [64]. In addition, decreased expression of FAK was found to upregulate the Wnt pathway. Through these mechanisms, significant enhancement of angiogenesis and re‐epithelialization by MIBs was observed. Subsequently, CD31 immunofluorescence staining was performed to assess vascular regeneration (Figure S15A). The MIB‐S group exhibited the most pronounced vascular regeneration, with a blood vessel density (∼22/mm^2^) that was 1.9‐fold and 1.6‐fold higher than that observed in the commercial and suture groups, respectively (Figure S15B). This enhanced angiogenesis can be attributed to the wound tension modulation exerted by the MIB‐S, which has been demonstrated to significantly influence the formation of the microvascular network [65]. Finally, in the remodeling phase, the reduced FAK expression led to the decreased expression of *Mmp3*, *Jun*, and *Ccn1*, indicating the downregulation of the TGF‐β pathway [66]. This hypothesis was further confirmed by immunofluorescence staining of TGF‐β, in which significantly attenuated TGF‐β signaling intensity was observed in the MIB‐S group (approximately 71% reduction compared to the MIB‐NS group) (Figure S16A,C). In addition, α‐SMA staining was performed to evaluate myofibroblast differentiation, which is closely associated with fibrotic tissue remodeling (Figure S16B) [67]. Significant inhibition of α‐SMA expression was observed in the MIB‐S group, indicating a balanced matrix degradation and deposition during tissue remodeling (Figure S16D). To further validate whether this regulation could be driven by tension cues rather than secondary in vivo consequences, we performed a strain‐programmed cell‐laden 3D collagen assay that mimics clinically relevant tension conditions [68, 69]. A constant 10% strain was used to represent physiological skin tension, while a constant 20% strain simulated the elevated wound tension observed under pathophysiological conditions (10%–20% strain). Additionally, a stepwise strain program (10%–20%–10% strain) was applied as a tension‐modulation regimen enabled by our MIBs. In NIH 3T3‐laden constructs (a fibroblast model designed to study inflammatory signaling and myofibroblast differentiation), the application of sustained 20% strain resulted in increased expression of FAK, NF‐κB, and α‐SMA markers. Conversely, a tension‐unloading trajectory led to reduced FAK and NF‐κB signals, as well as attenuated α‐SMA expression. These findings indicated that tension modulation can regulate mechanotransduction, as well as subsequent inflammatory signaling and myofibroblast differentiation (Figure S17). In HaCaT‐laden constructs (a keratinocyte model designed to probe epithelial junction maintenance and re‐epithelialization), the same trajectory was accompanied by the recovery of N‐cadherin (N‐Cad), supporting improved cell‐cell junction integrity under mechanically normalized conditions (Figure S18). In conclusion, it was found that the MIB, through its wound tension modulation capabilities, effectively reduces FAK expression, which in turn comprehensively regulates wound healing progression pathways including the NF‐κB, Wnt, and TGF‐β pathways, thereby enabling enhanced wound regeneration (Figure 5F).

### 2.6 Enhanced Wound Healing in Full‐Thickness Porcine Skin Wound Model

**FIGURE 6:**
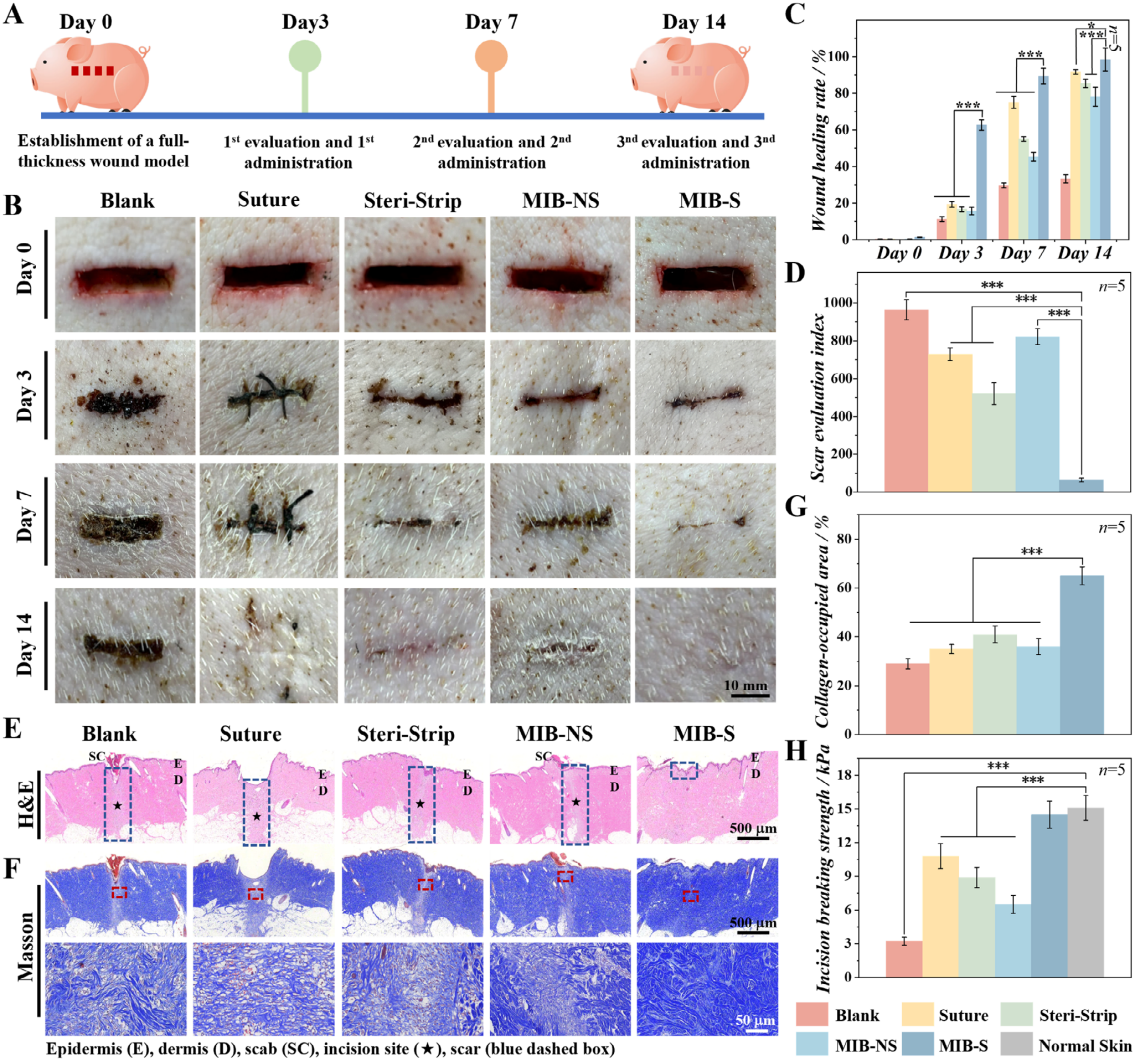
MIBs accelerated wound healing in a full‐thickness porcine skin wound model. (A) Schematic illustrating the establishment of the dorsal full‐thickness porcine skin wound model. (B) Representative wound photographs and corresponding healing progression maps under various treatments. (C) Quantitative analysis of wound healing rates among different groups. (D) Quantification of the Scar Elevation Index (SEI) of the healed tissue on day 14. (E) H&E‐stained images of wound tissue on day 14, with blue box indicating scar depth. (F) Masson's trichrome‐stained images of the wounds on day 14, with enlarged views of highlighted regions shown in the second row. Quantitative evaluation of (G) collagen content and (H) incision breaking strength in the healed tissue. All experiments were conducted with a sample size of *n* = 5 and analyzed using a one‐way or two‐way ANOVA followed by Tukey's post hoc test for multiple comparisons. Data are expressed as mean ± SD, with statistical significance denoted as *p < 0.05, and ***p < 0.001.

To enhance clinical relevance, we utilized a full‐thickness porcine skin defect model, as porcine skin closely resembles human skin in terms of structure, thickness, and healing characteristics. This model provides a clinically relevant platform to assess the translational potential of our MIBs, particularly with respect to scar formation and tissue remodeling (Figure 6A) [58]. The experimental group employed MIBs with a consistent 30% pre‐strain at all time points (designated as MIB‐S), a parameter selected based on its demonstrated ability to achieve robust wound closure in the porcine model and to provide a standardized contractile force, enabling direct comparison of wound healing progression across groups and time points. The non‐strained MIB group (designated as MIB‐NS) served as a control to specifically evaluate the role of contractile forces in the wound healing process. The MIB‐S group exhibited markedly accelerated wound closure compared to the other groups. Macroscopic images revealed uniform closure as early as day 7, with complete wound repair achieved by day 14, while residual unhealed areas persisted in the contractile force‐free MIB‐NS group and other groups (Figure 6B). Quantitative wound healing rate confirmed the superior performance of MIB‐S, demonstrating healing rates over 1.2 and 1.3 times faster than those of the Steri‐Strip and MIB‐NS groups, respectively (Figure 6C). Then, scar severity was assessed using the Scar Elevation Index (SEI), a clinically relevant metric that measures the ratio of scar area to underlying dermis area (Figure 6D) [70]. The MIB‐S group demonstrated a significantly lower SEI compared to the MIB‐NS group, highlighting its ability to mitigate excessive scar formation. These effects likely stem from the tension modulation exerted by the MIB‐S, which supports functional and aesthetic wound repair. To further investigate healing progression, histological evaluations were performed using H&E staining and Masson trichrome staining. By day 14, the MIB‐S group exhibited significantly complete epidermal regeneration, with the smallest wound gap observed among all groups (Figure 6E). Notably, complete wound closure and scar‐free healing were achieved exclusively in the MIB‐S group. Masson trichrome staining revealed superior collagen deposition in the MIB‐S‐treated wounds, characterized by highly organized and densely packed collagen fibers, indicative of optimized extracellular matrix reconstruction (Figure 6F,G). Additionally, the mechanical integrity of the newly formed tissue was evaluated through an incision breaking strength test (Figure 6H). The MIB‐S group demonstrated the highest breaking strength (∼14 kPa), exceeding that of the Steri‐Strip and MIB‐NS groups by 1.6‐fold and 2.2‐fold, respectively. This result underscores the enhanced durability and structural integrity of the regenerated tissue, which closely approximated the mechanical properties of normal skin.

Collectively, these findings demonstrate that MIB‐S, with its capacity for wound tension regulation, can accelerate wound healing by promoting re‐epithelialization, granulation tissue formation, and collagen deposition, while preserving tissue strength and suppressing scar formation, highlighting its significant translational potential.

## 3 Conclusion

In this study, we developed a bioinspired mechano‐intelligent Janus elastic bandage (MIB) that dynamically synchronizes adhesion and contraction to achieve scar‐attenuated wound healing. Inspired by the gecko locomotion mechanism, the MIB incorporates a Janus architecture with a hydrophilic, biomimetic wedged interior and a hydrophobic exterior. Fabricated through precision micromolding of fluid, photocrosslinkable poly(lactide–co–propylene glycol–co–lactide) dimethacrylates (PmLnD), the MIB amplifies van der Waals interactions and interfacial friction via its wedged interior, achieving robust tissue adhesion. Simultaneously, its pre‐strained elastic structure dynamically generates contractile forces. This synchronization of adhesion and contraction ensures effective mechanical force transmission, promotes precise wound closure, balances wound tension, and accelerates re‐epithelialization and angiogenesis while also mitigating inflammation. Mechanistically, the MIB downregulates FAK expression and modulates key wound healing pathways, including NF‐κB, Wnt, and TGF‐β, enabling phased healing progression and minimizing scar formation. We have created a mock‐up of the Instructions for Use (IFU) to provide clear guidance for clinicians on achieving the target pre‐strain and ensuring the effective application of the MIBs (Details in SI). The MIB is primarily positioned for incision‐type wound management, with efficacy demonstrated in acute rat and porcine models. A porcine‐skin circular‐opening test suggests feasibility on round geometries, but the current design is not intended for large‐area tissue‐loss wounds that require alternative wound‐care strategies. We acknowledge that the exclusive use of acute wound models is a meaningful limitation, as the most pressing clinical need lies in chronic wounds such as diabetic foot ulcers, which involve a distinct pathophysiology characterized by persistent inflammation, impaired fibroblast contractility, and dysregulated mechanotransduction. These are the conditions under which externally applied, programmable contractile forces may offer the greatest therapeutic benefit, yet also where the demands on the material and biological design are most stringent. Future work will therefore evaluate the MIB in established chronic wound models, including streptozotocin‐induced diabetic models, and explore targeted modifications such as pro‐regenerative surface functionalization, proangiogenic agent integration, and pre‐strain optimization to match the altered tissue mechanics of chronically wounded tissue. Altogether, by integrating mechanical and biochemical modulation within a single bioinspired elastic platform, the MIB represents a substantive advance in mechano‐active wound care, and addressing the chronic wound gap identified here constitutes the most important direction for its future translational development.

## 4 Experimental Section

### 4.1 Synthesis, Characterization, and Mechanical Evaluation of PmLnD

We synthesized a series of poly(lactide–co–propylene glycol–co–lactide) derivatives (PmLnD) using previously established protocols, selecting P68L8D as a representative example for synthesis process [36, 59]. The synthesis of P68L8D began with 400 g of propylene glycol (Macklin, China) reacting with 115.2 g of lactide (Macklin, China) to produce the intermediate poly(lactide–co–propylene glycol–co–lactide). Subsequently, 39.1 mL of methacryloyl chloride (Sigma‐Aldrich, Hong Kong) and 55.6 mL of triethylamine (Sigma‐Aldrich, Hong Kong) diluted in dichloromethane (Macklin, China) were added to the reaction system. To obtain pure PmLnD, the crude reaction mixture was purified as follows. First, the mixture was filtered through a double layer of filter paper using a Brinell funnel to remove large particulate impurities. The filtrate was then adjusted to neutral pH using triethylamine (TEA) and subjected to an additional vacuum filtration. The resulting filtrate was concentrated by rotary evaporation, and the concentrated residue was further dried. The dried material was subsequently treated with methyl tert‐butyl ether (MTBE) to induce precipitation, and the precipitate was removed to facilitate the removal of residual TEA, methacryloyl chloride (MAC), and dichloromethane (DCM). The clarified solution was then concentrated, diluted five‐fold with MTBE, and stored overnight at 4°C to promote further separation. For column purification, a silica gel column was prepacked and allowed to settle naturally for one day. The MTBE‐diluted crude product was loaded onto the silica gel column and eluted with MTBE. The initial MTBE eluent was discarded to remove remaining small‐molecule impurities. Once the product fraction began to elute, the corresponding fractions were collected. Finally, the collected solution was concentrated by rotary evaporation to obtain purified PmLnD.

The final product was characterized using Fourier‐transform infrared spectroscopy (FTIR, Bruker, USA) and ^1^H nuclear magnetic resonance (^1^H NMR, Jeol, Japan). Rheological analysis of PmLnD was conducted using a rheometer (Anton Paar, Austria) in rotational mode, measuring viscosity at shear rates ranging from 1 to 100/s. The mechanical properties of PmLnD were assessed using a universal testing machine (MTS, USA) in accordance with ASTM D638 standards [71]. Samples (20 × 5 mm) were subjected to tensile testing at a rate of 1 mm/min until failure at room temperature. The water contact angle (WCA) of PmLnD was measured using the sessile drop method with a contact angle analysis system (Krüss, Germany), applying 10 µL of deionized (DI) water at room temperature.

### 4.2 Segment Length and Methacrylation Efficiency (DM) Calculation

The resonance at ∼3.56 ppm (a,b) was assigned to the PPG backbone protons (3H per PPG repeat unit), thus *m* = ∫_3.56_/3. The signal at ∼1.58 ppm (c) corresponds to LA methyl protons. Since LA blocks are present on both ends, the total methyl contribution is 6H per Ln, thus *n* = ∫_1.58_/6. The vinyl protons of methacrylate appear at ∼6.21 ppm (e) and ∼5.60 ppm (d). For a dimethacrylated chain, each vinyl resonance corresponds to 2H in total. The methacrylation efficiency was calculated as:

(1) $$\mathrm{DM}\;(\%)=\frac{\left(\frac{\int \;6.21+\int \;5.60}{2}\right)}{2}\times 100\%$$

### 4.3 Fabrication, Surface Modification, and Characterization of the MIBs

Master molds of the MIBs with pre‐designed parameters were first fabricated based on copper alloy by machining. Next, a 10:1 PDMS solution created by PDMS prepolymer and curing agent (Sylgard 184, Dow Chemical Company, USA) was cast into the copper positive mold and degassed for 2 h at room temperature then cured at 80°C for at least 6 h. The PDMS negative mold was then obtained after curing and separation from the machined copper positive mold [37]. The precrosslinked P68L8D precursor was created by a supplementation of 4.5 wt.% hydroxyethyl methacrylate (HEMA, Sigma‐Aldrich, Hong Kong) and 0.5 wt.% phenylbis(2,4,6‐trimethylbenzoyl) phosphine oxide (Irgacure 819, BASF, Germany) in pure P68L8D [36]. To create the MIBs, all precursor solutions were sterilized by filtration through a 0.22 µm filter, and the MIB patches were fabricated in a sterile environment using the sterilized precursor. Briefly, photocrosslinkable P68L8D precursor was dispensed into the PDMS negative mold and degassed for at least 5 min at room temperature to remove bubbles, followed by photocrosslinking under 405 nm blue light for 2 min. After fabrication, the interior (tissue‐contacting) surface was treated with oxygen plasma using an atmospheric‐pressure plasma system for 5 min at 50 W to enhance surface hydrophilicity by introducing polar functional groups. The patches were vacuum‐sealed and stored at 4°C until use. For terminal sterilization, the outer packaging was irradiated with UV light for 30 min [72]. The morphology of the MIBs was examined using an optical microscope (Nikon, Japan) and a scanning electron microscope (SEM, Tescan, VEGA3, Czech Republic). The wedge area of the MIB was plasma‐treated to modify its surface properties, which were subsequently characterized using FTIR and WCA measurements to analyze the surface's chemical composition and wettability. Cyclic tensile tests involved stretching the bandages to a maximum tensile strain of 40% at 2 mm/min and then allowing relaxation for recovery.

### 4.4 In Vitro Biocompatibility Evaluation of the MIBs

L929 fibroblasts (ATCC, USA) were used to evaluate the in vitro biocompatibility of MIBs following ISO 10993 standards [42]. Briefly, samples (3 × 3 mm) were immersed in 1 mL Dulbecco's Modified Eagle's Medium (DMEM, Gibco, Hong Kong) for 24 h to produce conditioned medium. Subsequently, 3 × 10^4^ cells were seeded in 24‐well plates and cultured in the conditioned medium. Cell morphology was observed through bright‐field imaging at different time points. To assess cell viability and proliferation, Live/Dead Assay Kit and PicoGreen Assay Kit (Thermo Fisher, Hong Kong) were utilized, ensuring a comprehensive evaluation of biocompatibility [73].

### 4.5 Evaluation of Skin Irritation Potential of the MIBs

The MIBs animal experiment adhered to ISO 10993 standards [42]. The backs of five New Zealand White rabbits (male, 2.0–3.0 kg) were divided into three 2.5 × 2.5 cm areas for the skin irritation test with permission from the Ethics Committee of the Hong Kong Polytechnic University (Approval No. 22–23/331‐BME‐R‐OTHERS). The samples were divided into five groups: (1) negative control (thin cotton cloth), (2) positive control (cloth rinsed with saturated sodium dodecyl sulfate solution), (3) suture, (4) Steri‐strip, and (5) MIBs. Briefly, the rabbits’ backs were shaved 24 h before the application of each sample. Three samples from each group were placed on the skin beneath 2.5 × 2.5 cm gauze pads, which were secured with semi‐occlusive sterile medical dressings. After 24 h of direct contact with shaved skin, the materials were removed, and the test area was rinsed with lukewarm water. The skin was examined for visible changes such as erythema, at 0 (initial), 24‐, and 72‐hours post‐application. The mean erythema scores (MES) were assessed on a 0–4 scale, where 0 indicates no erythema, 1 represents slight erythema, 2 moderate erythema, 3 moderate to severe erythema, and 4 severe erythema.

### 4.6 Evaluation of Antifouling, Antibacterial Properties, and Moisture Retention of the MIBs

The MIBs were evaluated for their moisture retention, breathability, antifouling, and antibacterial properties. Antifouling properties were tested using fluorescein isothiocyanate‐labeled bovine serum albumin (FITC‐BSA) as a model protein [74]. Round samples with a radius of 0.8 mm were incubated with a 0.5 mg/mL FITC‐BSA solution, rinsed with PBS, and then examined for fluorescence signals using a fluorescence microscope (Nikon, Japan). The antibacterial properties were tested against Gram‐negative Escherichia coli (*E. coli*, ATCC) and Gram‐positive Staphylococcus aureus (*S. aureus*, ATCC) [75]. Bacterial suspension at a concentration of 1 × 10^5^ CFU/mL was incubated with the materials at 37°C for 48 h. Post‐incubation, materials were gently washed with phosphate‐buffered saline (PBS) to remove planktonic bacteria. To determine total bacterial count, samples were vortexed in 1 mL of PBS, serially diluted (1:10000), and plated on Lysogeny Broth (LB) agar plates, which were incubated at 37°C for 24 h. Colony‐forming units (CFUs) were counted to quantify bacterial load. For the live/dead staining assay, samples were incubated with 200 µL of staining solution containing 1 µM SYTO 9 and 5 µM Propidium Iodide (PI) in PBS for 45 min at room temperature. After washing with PBS, samples were observed under a fluorescence microscope (Zeiss, Germany) to assess bacterial viability. The water vapor transmission rate (WVTR) of the samples was measured following the previous protocol [76]. Briefly, round samples of 30 mm diameter were used to seal the mouth of bottles with 29 mm diameter containing 20 mL DI water. The bottles were placed in an incubator at 37°C and 35% humidity. At different time points, water loss was weighed for the WVTR calculation. The swelling behavior was evaluated by immersing samples in deionized (DI) water and assessing their quality change by tensile testing, following established literature protocols [77].

### 4.7 Evaluation of Adhesion Strength, Contractile Force, and Non‐Invasive Skin Adhesion Properties of the MIBs

To assess the adhesion strength and contractile force of MIBs, a custom‐designed testing apparatus was employed based on an established protocol [78, 79]. The system incorporated a pulley mechanism connected to a calibrated load cell, enabling precise application of varying preloads. Preload ratios from 5% to 35% were applied to establish bonds between the MIBs (1 cm × 1 cm) and a rigid surface, with the magnitude of the preload adjusted through the pulley system. Adhesive failure was defined as the force at which the bond between the MIBs and the surface was disrupted. Closure strength was evaluated by applying tensile forces perpendicular to simulated wound edges, quantifying the bandage's capacity to generate contractile forces for effective wound closure. To precisely measure the contact area of the MIBs, glass substrates were utilized for visualization, with contact dynamics captured via stereomicroscopy and analyzed using digital imaging techniques. This integrated approach ensured a robust evaluation of adhesive strength, contractile force, and their relationship with the contact interface. Data were systematically collected and processed through the load cell and digital analysis system for accuracy and reliability.

The actual adhesion properties of MIBs were evaluated on various body regions (including shoulder, forearm, and knee) of an adult male, to examine their performance under different mechanical stresses and surface contours. The experiments involved applying MIBs to these specific body areas and observing their adhesion stability and contraction efficiency during natural movements. All procedures were ethically approved by the Ethics Committee of The Hong Kong Polytechnic University (Approval No. HSEARS20240509001). The informed written consent was obtained from the participant involved in the experiment. The non‐invasive adhesion properties of the MIBs were evaluated on the skin of one‐week‐old Sprague‐Dawley (SD) rats, approved by the Ethics Committee of The Hong Kong Polytechnic University (20–21/204‐BME‐R‐OTHERS) [54]. For the patch‐removal experiment, one‐week‐old rats were anesthetized with isoflurane, after which the MIBs were applied to the dorsal skin for 10 min and then gently removed in a single step. After patch removal, the animals were euthanized with CO_2_, and skin tissues from the treated area were harvested for gross observation and H&E staining. This assay was designed as a brief, non‐surgical removal test, and no wound creation or repeated stripping was involved. Commercial Hansaplast polyurethane bandages were used as a control group and subjected to the same testing procedure.

### 4.8 Finite Element Simulation of Stress Distribution and Mechanical Behavior of the MIBs

A two‐dimensional axisymmetric finite element model was developed in COMSOL Multiphysics to represent a circular wound region surrounded by skin and covered by a pre‐strained dressing (MIB). The skin's hyperelastic behavior at large strains was captured using a second‐order Ogden model, whose strain energy density is given by

(2) $$W=\sum_{i=1}^{2}\;\frac{2\mu_{i}}{\alpha_{i}^{2}}\;\;\left(\;\lambda_{1}^{\alpha_{i}}+\lambda_{2}^{\alpha_{i}}+\lambda_{3}^{\alpha_{i}}-3\right)$$

where µi and αi are material constants determined through nonlinear curve fitting of experimental tensile data, and λ_1_, λ_2_, λ_3_ are the principal stretch ratios. The dressing was modeled with a Neo‐Hookean hyperelastic formulation, expressed as

(3) $$W=\frac{\mu }{2}\;\left(\;\bar{\lambda_{1}^{2}}+\bar{\lambda_{2}^{2}}+\bar{\lambda_{3}^{2}}-3\right)$$

where µ is the shear modulus and $\bar{\lambda_{i}}$ are the deviatoric principal stretches. The far‐field skin boundary at *r*  = *r_out_* was constrained to mimic fixation by surrounding tissue. Contact between the dressing and skin was modeled using a frictional contact pair, allowing separation while preventing interpenetration. The domain was discretized using second‐order elements with local mesh refinement at the wound edge and the skin–dressing interface to capture stress gradients. A stationary nonlinear solver was then used to compute the von Mises stress distribution, with a particular focus on circumferential (hoop) stress around the wound edge. The resulting contour plots and numerical data at critical regions were compared across varying pre‐strain levels to elucidate how the MIB modulates stress fields during wound closure.

### 4.9 In Vivo Evaluation of Wound Healing Efficacy Using a Full‐Thickness Rat Skin Wound Model

The in vivo efficacy of MIBs was evaluated using a full‐thickness skin wound model in rats, approved by the Ethics Committee of The Hong Kong Polytechnic University (Approval No. 23–24/733‐ABCT‐R‐GRF). Sprague‐Dawley rats (male, weight: 200–220 g, *n* = 4) were anesthetized with 1% pentobarbital [80, 81]. The principle of selection of experimental animals was generated by an online random number generator. The dorsal skin was exposed and disinfected, and two linear full‐thickness wounds (2 cm in length) were created on the back of each rat. The wounds were treated with different materials, including unstretched (MIB‐NS) and 20% pre‐strain (MIB‐S) MIBs, sutures, and commercial Steri‐Strips. For the MIB‐NS group, the bandages were fixed in place using a standard medical adhesive tape (3M Micropore), which is highly breathable and does not affect wound healing outcomes. This approach ensured stable coverage throughout the healing period without impacting the experimental results. At specific time points (3, 7, and 14 days), equidistant photographs of the healing wounds were taken to assess wound closure. Wound healing rate depicting the percentage reduction in wound surface area over time was calculated by the formula: Wound healing rate (%) = [(initial wound area – wound area at each time point)/initial wound area] × 100% [82]. For histological analysis, wound samples were collected at 7‐ and 14‐days post‐treatment. Four rats from each group were euthanized, and wound samples were fixed in formalin (TissuePro, Hong Kong). The samples were then embedded in paraffin, sectioned, and stained with Hematoxylin and Eosin (H&E) to evaluate the wound healing process, and with Masson's Trichrome to determine collagen deposition [59].

For the immunofluorescence staining, the percentage of positive area was calculated as the proportion of the tissue section exhibiting specific immunofluorescence signal for a given marker (e.g., Col‐I or Col‐III) relative to the total area of the tissue section analyzed [83]. Incision breaking strength was measured using an Instron universal testing machine. Full‐thickness skin strips (4 × 1 cm) were carefully dissected, with a 1 cm^2^ section clamped on each side of the wound. The test was run with a tensile speed of 10 mm/min [84]. The breaking strength was recorded as the load at failure. Then, Col‐I and Col‐III immunofluorescence staining were additionally performed to evaluate the scar formation [58].

### 4.10 Transcriptomic Evaluation of the MIBs for Wound Healing

On day 7, wound tissue samples were collected for RNA sequencing to analyze the transcriptome. Total RNA was extracted from the skin tissues using TRIzol reagent (Thermo Fisher, Hong Kong). RNA sequencing was performed using the Novaseq 6000 platform (Illumina, USA). Differentially expressed mRNAs were identified using the edgeR R package, with a fold change >2 or <0.5 for paired comparisons. Gene Ontology (GO) enrichment analysis and KEGG pathway enrichment analysis were conducted using the OmicStudio tools. Immunofluorescence staining for FAK, CD31, TGF‐β, α‐SMA, iNOS, CD68, and CD163 was performed following previously established methods [59, 85, 86]. The primers of tested genes were listed in Table S3.

### 4.11 In Vitro Strain‐Programmed 3D Collagen Model And Immunofluorescence Analysis

A cell‐laden 3D collagen model was established by embedding NIH 3T3 fibroblasts (0.5 × 10^6^ cells/cm^3^) or HaCaT keratinocytes (2 × 10^6^ cells/cm^3^) in type I collagen gels cast in a custom mold with sponge anchors at both ends to enable uniaxial mechanical constraint. After gelation and 24 h pre‐culture under constrained conditions, constructs were subjected to programmed tensile strain to mimic clinically relevant tension states, including constant 10% strain (physiological skin tension), constant 20% strain (elevated wound tension), and a stepwise strain program (10%–20%–10% strain) as an MIB‐relevant tension‐unloading trajectory. Following stimulation, samples were fixed, permeabilized, and immunostained. FAK, NF‐κB, and α‐SMA were analyzed in NIH 3T3‐laden constructs, whereas N‐cadherin was analyzed in HaCaT‐laden constructs. Confocal z‐stack images were acquired using identical settings across groups, and the positive area percentage was quantified using the same thresholding criteria for all groups.

### 4.12 In Vivo Evaluation of Wound Healing and Scar Quality Using a Full‐Thickness Porcine Skin Wound Model

The porcine model using Panamanian mini pigs (male, weight: 12–15 kg, *n* = 5) was established by Huateng Biotechnology Co., Ltd. (Guangzhou, China), with ethical approval (Approval No. B202401‐17). All procedures were performed by qualified veterinarians under strict aseptic conditions. The principle of selection of experimental animals was generated by an online random number generator. Prior to surgery, the hair on the pigs’ backs were completely shaved, and the skin was disinfected with ethanol. Surgeries were conducted under general anesthesia. Five full‐thickness wounds (2.5 cm × 0.5 cm) were created on each side of the spine using a double‐sided scalpel, maintaining a 4 cm gap between each wound. The wounds were applied with different treatment, including MIB‐NS, MIB‐S, sutures, and commercial Steri‐Strip. Dressings were applied three times at defined intervals (days 3, 7, and 14) to closely mimic clinical practice, where wound dressings are regularly changed to maintain hygiene, monitor healing progression, and reduce the risk of infection. Postoperatively, each pig was housed individually and monitored daily. Wound images were captured, and wound sizes were measured at predetermined time points. On day 14, the pigs were euthanized, and wound tissues were collected for histological analysis using H&E and Masson's Trichrome staining to evaluate the microstructure of the regenerated tissue and collagen deposition. Scar quality was assessed using a standardized evaluation method combining clinical photography and expert scoring based on the Visual Analogue Score (VAS) [87]. High‐resolution images of the scars were captured under consistent lighting conditions. Three experienced assessors, blinded to the treatment groups, independently scored the images. Each assessor marked a 100‐point grading scale, where 0 represented unwounded skin and 100 indicated severe scarring (raised, hyperpigmented, or erythematous). The average score across assessors provided a robust and reproducible measure of scar severity. The incision breaking strength was tested using the same method as described earlier.

### 4.13 Statistical Analysis

Experiments are performed in triplicate unless indicated, and all data demonstrated consists of the mean and standard deviation (SD). Statistical differences are analyzed using one‐ or two‐way analysis of variance (ANOVA) along with Turkey's multiple comparisons test. *p < 0.05, **p < 0.01, ***p < 0.001 are considered statistically significant.

## Conflicts of Interest

The authors declare no conflicts of interest.

## Supporting information




**Supporting File 1**: adma73347‐sup‐0001‐SuppMat.docx.

**Supporting File 2**: adma73347‐sup‐0002‐VideoS1‐S3.zip.

## Data Availability

The data that support the findings of this study are available from the corresponding author upon reasonable request.

## References

[1] C. Wang , E. Shirzaei Sani , C.‐D. Shih , et al., “Wound Management Materials And Technologies From Bench to Bedside and Beyond,” Nature Reviews Materials 9 (2024): 550–566, 10.1038/s41578-024-00693-y. PMC12176411 40535534

[2] B. R. Freedman , C. Hwang , S. Talbot , B. Hibler , S. Matoori , and D. J. Mooney , “Breakthrough Treatments for Accelerated Wound Healing,” Science Advances 9 (2023): ade7007, 10.1126/sciadv.ade7007. PMC10191440 37196080

[3] L. Wang , F. Wan , Y. Xu , et al., “Hierarchical Helical Carbon Nanotube Fibre as a Bone‐integrating Anterior Cruciate Ligament Replacement,” Nature Nanotechnology 18 (2023): 1085–1093, 10.1038/s41565-023-01394-3. 37142709

[4] Y. Liang , J. He , and B. Guo , “Functional Hydrogels as Wound Dressing to Enhance Wound Healing,” ACS Nano 15 (2021): 12687–12722, 10.1021/acsnano.1c04206. 34374515

[5] Z. Wang , L. Xiang , F. Lin , Y. Tang , L. Deng , and W. Cui , “A Biomaterial‐Based Hedging Immune Strategy for Scarless Tendon Healing,” Advanced Materials 34 (2022): 2200789, 10.1002/adma.202200789. 35267215

[6] S. Blacklow , J. Li , B. R. Freedman , M. Zeidi , C. Chen , and D. J. Mooney , “Bioinspired Mechanically Active Adhesive Dressings to Accelerate Wound Closure,” Science Advances 5 (2019): aaw3963, 10.1126/sciadv.aaw3963. PMC6656537 31355332

[7] A. P. M. Nozaki , M. H. de Melo Lima , and Â. M. Moraes , “Sprayable Bioactive Dressings for Skin Wounds: Recent Developments and Future Prospects,” Biomedical Materials & Devices 1 (2023): 569–586, 10.1007/s44174-022-00047-8.

[8] C. A. J. Shapiro , M. R. C. Dinsmore , and L. J. H. North Jr , “Tensile Strength of Wound Closure With Cyanoacrylate Glue,” American Surgeon 67 (2001): 1113–1115, 10.1177/000313480106701118. 11730233

[9] G. Theocharidis , H. Yuk , H. Roh , et al., “A Strain‐programmed Patch for the Healing of Diabetic Wounds,” Nature Biomedical Engineering 6 (2022): 1118–1133, 10.1038/s41551-022-00905-2. 35788686

[10] H. Chen , R. Zhang , G. Zhang , et al., “Naturally Inspired Tree‐Ring Structured Dressing Provides Sustained Wound Tightening and Accelerates Closure,” Advanced Materials 37 (2025): 2410845, 10.1002/adma.202410845. 39533478

[11] J. Sun , W. Jia , H. Qi , et al., “An Antioxidative and Active Shrinkage Hydrogel Integratedly Promotes Re‐Epithelization and Skin Constriction for Enhancing Wound Closure,” Advanced Materials 36 (2024): 2312440, 10.1002/adma.202312440. 38332741

[12] J. Hu , T. Wei , H. Zhao , and M. Chen , “Mechanically Active Adhesive and Immune Regulative Dressings for Wound Closure,” Matter 4 (2021): 2985–3000, 10.1016/j.matt.2021.06.044.

[13] J. Yi , G. Zou , J. Huang , et al., “Water‐responsive Supercontractile Polymer Films for Bioelectronic Interfaces,” Nature 624 (2023): 295–302, 10.1038/s41586-023-06732-y. 38092907

[14] X. Meng , X. Xiao , S. Jeon , et al., “Self‐contracting, Battery‐free Triboelectric Wound Healing Strip With Strong Wet Adhesion,” Nature Communications 16 (2025): 7220, 10.1038/s41467-025-62312-w. PMC12325624 40764479

[15] Z. Zheng , X. Chen , Y. Wang , et al., “Self‐Growing Hydrogel Bioadhesives for Chronic Wound Management,” Advanced Materials 36 (2024): 2408538, 10.1002/adma.202408538. 39149779

[16] Y. Wang , Z. Li , C. Zhang , Z. Jin , and A. C. Midgley , “Dermal Fibrosis and the Current Scope of Hydrogel Strategies for Scarless Wound Healing,” Fibrosis 2 (2024): 10010, 10.70322/fibrosis.2024.10010.

[17] F. Boccafoschi , M. Bosetti , S. Gatti , and M. Cannas , “Dynamic Fibroblast Cultures,” Cell Adhesion & Migration 1 (2007): 124–128, 10.4161/cam.1.3.5144. 19262127 PMC2634011

[18] J. L. Balestrini and K. L. Billiar , “Magnitude and Duration of Stretch Modulate Fibroblast Remodeling,” Journal of Biomechanical Engineering 131 (2009): 051005, 10.1115/1.3049527. 19388775

[19] L. Chang , H. Du , F. Xu , C. Xu , and H. Liu , “Hydrogel‐enabled Mechanically Active Wound Dressings,” Trends in Biotechnology 42 (2024): 31–42, 10.1016/j.tibtech.2023.06.004. 37453911

[20] H. Yuk , C. E. Varela , C. S. Nabzdyk , et al., “Dry Double‐sided Tape for Adhesion of Wet Tissues and Devices,” Nature 575 (2019): 169–174, 10.1038/s41586-019-1710-5. 31666696

[21] J. L. Daristotle , S. T. Zaki , L. W. Lau , et al., “Pressure‐sensitive Tissue Adhesion and Biodegradation of Viscoelastic Polymer Blends,” ACS Applied Materials & Interfaces 12 (2020): 16050–16057, 10.1021/acsami.0c00497. 32191429 PMC7271901

[22] C. Cai , H. Zhu , Y. Chen , et al., “Mechanoactive Nanocomposite Hydrogel to Accelerate Wound Repair in Movable Parts,” ACS Nano 16 (2022): 20044–20056, 10.1021/acsnano.2c07483. 36300517

[23] W. Feng and Z. Wang , “Tailoring the Swelling‐Shrinkable Behavior of Hydrogels for Biomedical Applications,” Advanced Science 10 (2023): 2303326, 10.1002/advs.202303326. 37544909 PMC10558674

[24] Q. Wang , W. Zheng , J. Wang , et al., “Biomimetic Dual‐layer Architectural Hydrogel Bandage With Smart Thermally Self‐contraction for Enhanced Wound Closure and Burn Wound Healing,” ACS Applied Materials & Interfaces 17 (2025): 30747–30758, 10.1021/acsami.5c06512. 40386938

[25] R. Dong and B. Guo , “Smart Wound Dressings for Wound Healing,” Nano Today 41 (2021): 101290, 10.1016/j.nantod.2021.101290.

[26] J. Rosińczuk , J. Taradaj , R. Dymarek , and M. Sopel , “Mechanoregulation of Wound Healing and Skin Homeostasis,” Chronic Wounds, Wound Dressings and Wound Healing (Springer, 2018), 10.1007/15695_2017_107. PMC4931093 27413744

[27] S. J. Frost , D. Mawad , J. Hook , and A. Lauto , “Micro‐ and Nanostructured Biomaterials for Sutureless Tissue Repair,” Advanced Healthcare Materials 5 (2016): 401–414, 10.1002/adhm.201500589. 26725593

[28] L. Wang , Y. Hui , C. Fu , Z. Wang , M. Zhang , and T. Zhang , “Recent Advances in Gecko‐inspired Adhesive Materials and Application,” Journal of Adhesion Science and Technology 34 (2020): 2275–2291, 10.1080/01694243.2020.1760478.

[29] S. J. Wu and X. Zhao , “Bioadhesive Technology Platforms,” Chemical Reviews 123 (2023): 14084–14118, 10.1021/acs.chemrev.3c00380. 37972301

[30] A. Mahdavi , L. Ferreira , C. Sundback , et al., “A Biodegradable and Biocompatible Gecko‐inspired Tissue Adhesive,” Proceedings of the National Academy of Sciences 105 (2008): 2307–2312, 10.1073/pnas.0712117105. PMC2268132 18287082

[31] P. Chansoria , A. Chaudhari , E. L. Etter , et al., “Instantly Adhesive and Ultra‐elastic Patches for Dynamic Organ and Wound Repair,” Nature Communications 15 (2024): 4720, 10.1038/s41467-024-48980-0. PMC11148085 38830847

[32] G. Choi , J. Kim , H. Kim , et al., “Motion‐Adaptive Tessellated Skin Patches With Switchable Adhesion for Wearable Electronics,” Advanced Materials 37 (2025): 2412271, 10.1002/adma.202412271. 39428834 PMC11775872

[33] S. Basak , “Walking Through the Biomimetic Bandages Inspired by Gecko's Feet,” Bio‐Design and Manufacturing 3 (2020): 148–154, 10.1007/s42242-020-00069-5.

[34] X. Li , D. Tao , H. Lu , et al., “Recent Developments in gecko‐inspired Dry Adhesive Surfaces From Fabrication to Application,” Surface Topography: Metrology and Properties 7 (2019): 023001, 10.1088/2051-672X/ab1447.

[35] D. M. Fitzgerald , Y. L. Colson , and M. W. Grinstaff , “Advancing Pressure‐sensitive Adhesives for Internal Wound Closure,” Nature Reviews Materials 8 (2023): 3–5, 10.1038/s41578-022-00516-y. PMC10193518 37206300

[36] Y. Yang , Q. Zhang , T. Xu , et al., “Photocrosslinkable Nanocomposite Ink for Printing Strong, Biodegradable and Bioactive Bone Graft,” Biomaterials 263 (2020): 120378, 10.1016/j.biomaterials.2020.120378. 32932140

[37] Y. Yang , T. Xu , H. P. Bei , Y. Zhao , and X. Zhao , “Sculpting Bio‐Inspired Surface Textures: An Adhesive Janus Periosteum,” Advanced Functional Materials 31 (2021): 2104636, 10.1002/adfm.202104636.

[38] B. Shiroud Heidari , R. Ruan , E. Vahabli , et al., “Natural, Synthetic and Commercially‐available Biopolymers Used to Regenerate Tendons and Ligaments,” Bioactive Materials 19 (2023): 179–197, 10.1016/j.bioactmat.2022.04.003. 35510172 PMC9034322

[39] S. Jin , Y. Yu , T. Zhang , et al., “Surface Modification Strategies to Reinforce the Soft Tissue Seal at Transmucosal Region of Dental Implants,” Bioactive Materials 42: 404–432, 10.1016/j.bioactmat.2024.08.042. PMC11415887 39308548

[40] J. Yi , X. Ren , Y. Li , et al., “Rapid‐Response Water‐Shrink Films With High Output Work Density Based on Polyethylene Oxide and α‐Cyclodextrin for Autonomous Wound Closure,” Advanced Materials 36 (2024): 2403551, 10.1002/adma.202403551. 38837826

[41] E. Marin , F. Boschetto , and G. Pezzotti , “Biomaterials and Biocompatibility: An Historical Overview,” Journal of Biomedical Materials Research Part A 108 (2020): 1617–1633, 10.1002/jbm.a.36930. 32196949

[42] Z. Ma , Q. Huang , Q. Xu , et al., “Permeable Superelastic Liquid‐metal Fibre Mat Enables Biocompatible and Monolithic Stretchable Electronics,” Nature Materials 20 (2021): 859–868, 10.1038/s41563-020-00902-3. 33603185

[43] Y. Guo , Y. Wang , X. Zhao , et al., “Snake Extract–Laden Hemostatic Bioadhesive Gel Cross‐Linked By Visible Light,” Science Advances 7 (2021): abf9635, 10.1126/sciadv.abf9635. PMC8279511 34261653

[44] C. Jiang , G. Wang , R. Hein , N. Liu , X. Luo , and J. J. Davis , “Antifouling Strategies for Selective In Vitro and In Vivo Sensing,” Chemical Reviews 120 (2020): 3852–3889, 10.1021/acs.chemrev.9b00739. 32202761

[45] K. Y. Kwon , S. Cheeseman , A. Frias‐De‐Diego , et al., “A Liquid Metal Mediated Metallic Coating for Antimicrobial and Antiviral Fabrics,” Advanced Materials 33 (2021): 2104298, 10.1002/adma.202104298. 34550628

[46] N. Tang , R. Zhang , Y. Zheng , et al., “Highly Efficient Self‐Healing Multifunctional Dressing With Antibacterial Activity for Sutureless Wound Closure and Infected Wound Monitoring,” Advanced Materials 34 (2022): 2106842, 10.1002/adma.202106842. 34741350

[47] R. Xu , G. Luo , H. Xia , et al., “Novel Bilayer Wound Dressing Composed of Silicone Rubber With Particular Micropores Enhanced Wound Re‐epithelialization and Contraction,” Biomaterials 40 (2015): 1–11, 10.1016/j.biomaterials.2014.10.077. 25498800

[48] V. W. Wong , K. Levi , S. Akaishi , G. Schultz , and R. H. Dauskardt , “Scar Zones: Region‐Specific Differences in Skin Tension May Determine Incisional Scar Formation,” Plastic and Reconstructive Surgery 129 (2012), 1272–1276, 10.1097/PRS.0b013e31824eca79. 22634644

[49] Y. Tian , N. Pesika , H. Zeng , et al., “Adhesion and Friction in Gecko Toe Attachment and Detachment,” Proceedings of the National Academy of Sciences 103 (2006): 19320–19325, 10.1073/pnas.0608841103. PMC1748224 17148600

[50] D. Tao , X. Gao , H. Lu , et al., “Controllable Anisotropic Dry Adhesion in Vacuum: Gecko Inspired Wedged Surface Fabricated With Ultraprecision Diamond Cutting,” Advanced Functional Materials 27 (2017): 1606576, 10.1002/adfm.201606576.

[51] J. Yu , S. Chary , S. Das , et al., “Gecko‐Inspired Dry Adhesive for Robotic Applications,” Advanced Functional Materials 21 (2011): 3010–3018, 10.1002/adfm.201100493.

[52] Y. Sekiguchi , K. Takahashi , and C. Sato , “Adhesion Mechanism of a Gecko‐inspired Oblique Structure With an Adhesive Tip for Asymmetric Detachment,” Journal of Physics D: Applied Physics 48 (2015): 475301, 10.1088/0022-3727/48/47/475301.

[53] Z. Ma , G. Bao , and J. Li , “Multifaceted Design and Emerging Applications of Tissue Adhesives,” Advanced Materials 33 (2021): 2007663, 10.1002/adma.202007663. 33956371

[54] Y. Jiang , X. Zhang , W. Zhang , et al., “Infant Skin Friendly Adhesive Hydrogel Patch Activated at Body Temperature for Bioelectronics Securing and Diabetic Wound Healing,” ACS Nano 16 (2022): 8662–8676, 10.1021/acsnano.2c00662. 35549213

[55] W. Li , L. Jiang , S. Wu , et al., “A Shape‐Programmable Hierarchical Fibrous Membrane Composite System to Promote Wound Healing in Diabetic Patients,” Small 18 (2022): 2107544, 10.1002/smll.202107544. 35038225

[56] Y. Gao , X. Han , J. Chen , et al., “Hydrogel–mesh Composite For Wound Closure,” Proceedings of the National Academy of Sciences 118 (2021): 2103457118, 10.1073/pnas.2103457118. PMC8285977 34264848

[57] G. Yao , X. Mo , C. Yin , et al., “A Programmable and Skin Temperature–Activated Electromechanical Synergistic Dressing For Effective Wound Healing,” Science Advances 8 (2022): abl8379, 10.1126/sciadv.abl8379. PMC8791608 35080981

[58] J. Yang , X. Jin , W. Liu , and W. Wang , “A Programmable Oxygenation Device Facilitates Oxygen Generation and Replenishment to Promote Wound Healing,” Advanced Materials 35 (2023): 2305819, 10.1002/adma.202305819. 37695102

[59] Y. Yang , D. Suo , T. Xu , et al., “Sprayable Biomimetic Double Mask With Rapid Autophasing and Hierarchical Programming for Scarless Wound Healing,” Science Advances 10 (2024): ado9479, 10.1126/sciadv.ado9479. PMC11323895 39141725

[60] S. Y. Lee , S. Jeon , Y. W. Kwon , et al., “Combinatorial Wound Healing Therapy Using Adhesive Nanofibrous Membrane Equipped With Wearable LED Patches for Photobiomodulation,” Science Advances 8 (2022): abn1646, 10.1126/sciadv.abn1646. PMC9012471 35427152

[61] Y. Shen , G. Xu , H. Huang , et al., “Sequential Release of Small Extracellular Vesicles From Bilayered Thiolated Alginate/Polyethylene Glycol Diacrylate Hydrogels for Scarless Wound Healing,” ACS Nano 15 (2021): 6352–6368, 10.1021/acsnano.0c07714. 33723994

[62] S. Shan , B. Fang , Y. Zhang , et al., “Mechanical stretch promotes tumoricidal M1 polarization via the FAK/NF‐κB signaling pathway,” FASEB Journal 33 (2019): 13254–13266, 10.1096/fj.201900799RR. 31539281

[63] Y. Jia , J. Hu , K. An , et al., “Hydrogel Dressing Integrating FAK Inhibition and ROS Scavenging for Mechano‐chemical Treatment of Atopic Dermatitis,” Nature Communications 14 (2023): 2478, 10.1038/s41467-023-38209-x. PMC10148840 37120459

[64] M. H. Park , E. D. Lee , and W.‐J. Chae , “Macrophages and Wnts in Tissue Injury and Repair,” Cells 11 (3592): 3592, 10.3390/cells11223592. 36429021 PMC9688352

[65] M. A. Ruehle , E. A. Eastburn , S. A. LaBelle , et al., “Extracellular Matrix Compression Temporally Regulates Microvascular Angiogenesis,” Science Advances 6 (2020): abb6351, 10.1126/sciadv.abb6351. PMC7442478 32937368

[66] A. Leask , “Focal Adhesion Kinase: A Key Mediator of Transforming Growth Factor Beta Signaling in Fibroblasts,” Advances in Wound Care 2 (2013): 247–249, 10.1089/wound.2012.0363. 24527346 PMC3857352

[67] J. Davis , N. Salomonis , N. Ghearing , et al., “MBNL1‐mediated Regulation of Differentiation RNAs Promotes Myofibroblast Transformation and the Fibrotic Response,” Nature Communications 6 (2015): 10084, 10.1038/ncomms10084. PMC4703843 26670661

[68] K. Chen , S. H. Kwon , D. Henn , et al., “Disrupting Biological Sensors of Force Promotes Tissue Regeneration in Large Organisms,” Nature Communications 12 (2021): 5256, 10.1038/s41467-021-25410-z. PMC8421385 34489407

[69] Y. Hong , X. Peng , H. Yu , et al., “Cell–Matrix Feedback Controls Stretch‐Induced Cellular Memory And Fibroblast Activation,” Proceedings of the National Academy of Sciences 122 (2025): 2322762122, 10.1073/pnas.2322762122. PMC11962495 40100625

[70] J. Zhang , Y. Zheng , J. Lee , et al., “A Pulsatile Release Platform Based on Photo‐induced Imine‐crosslinking Hydrogel Promotes Scarless Wound Healing,” Nature Communications 12 (2021): 1670, 10.1038/s41467-021-21964-0. PMC7960722 33723267

[71] S. S. Raj , A. Kuzmin , K. Subramanian , S. Sathiamoorthyi , and K. T. Kandasamy , “Philosophy of Selecting ASTM Standards for Mechanical Characterization of Polymers and Polymer Composites,” Materiale Plastice 58 (2021), 247–256, 10.37358/MP.21.3.5523.

[72] B. Paosawatyanyong , K. Kamlangkla , and S. Hodak , “Hydrophobic and Hydrophilic Surface Nano‐modification of PET Fabric by Plasma Process,” Journal of Nanoscience and Nanotechnology 10 (2010): 7050–7054, 10.1166/jnn.2010.2849. 21137863

[73] P. Vázquez‐Aristizabal , M. Henriksen‐Lacey , C. García‐Astrain , et al., “Biofabrication and Monitoring of a 3D Printed Skin Model for Melanoma,” Advanced Healthcare Materials 13 (2024): 2401136, 10.1002/adhm.202401136. 38992996 PMC12344628

[74] Z. Lin , Y. Ma , C. Zhao , et al., “An Extremely Simple Method for Fabricating 3D Protein Microarrays With an Anti‐fouling Background and High Protein Capacity,” Lab on A Chip 14 (2014): 2505–2514, 10.1039/C4LC00223G. 24852169

[75] W. He , J. Bai , X. Chen , et al., “Reversible dougong structured receptor–ligand recognition for building dynamic extracellular matrix mimics,” Proceedings of the National Academy of Sciences 119 (2022): 2117221119, 10.1073/pnas.2117221119. PMC8872741 35181608

[76] M. C. Straccia , I. Romano , A. Oliva , G. Santagata , and P. Laurienzo , “Crosslinker Effects on Functional Properties of Alginate/N‐succinylchitosan Based Hydrogels,” Carbohydrate Polymers 108 (2014): 321–330, 10.1016/j.carbpol.2014.02.054. 24751280

[77] T. Cheng , F. Wang , Y.‐Z. Zhang , et al., “3D printable Conductive Polymer Hydrogels With Ultra‐high Conductivity and Superior Stretchability for Free‐standing Elastic all‐gel Supercapacitors,” Chemical Engineering Journal 450 (2022): 138311, 10.1016/j.cej.2022.138311.

[78] J. Seo , J. Eisenhaure , and S. Kim , “Micro‐wedge Array Surface of a Shape Memory Polymer as a Reversible Dry Adhesive,” Extreme Mechanics Letters 9 (2016): 207–214, 10.1016/j.eml.2016.07.007.

[79] A. Hajj‐Ahmad , A. K. Han , M. A. Lin , G. H. Glover , and M. R. Cutkosky , “An Electrostatically Actuated Gecko Adhesive Clutch,” Advanced Materials Technologies 8 (2023): 2202025, 10.1002/admt.202202025.

[80] X. Zhao , Q. Lang , L. Yildirimer , et al., “Photocrosslinkable Gelatin Hydrogel for Epidermal Tissue Engineering,” Advanced Healthcare Materials 5 (2016): 108–118, 10.1002/adhm.201500005. 25880725 PMC4608855

[81] Y. Liang , Z. Li , Y. Huang , R. Yu , and B. Guo , “Dual‐dynamic‐bond Cross‐linked Antibacterial Adhesive Hydrogel Sealants With on‐demand Removability for Post‐wound‐closure and Infected Wound Healing,” ACS Nano 15 (2021): 7078–7093, 10.1021/acsnano.1c00204. 33764740

[82] R. Yan , X. Zhang , H. Wang , et al., “Autonomous, Moisture‐Driven Flexible Electrogenerative Dressing for Enhanced Wound Healing,” Advanced Materials 37 (2025): 2418074, 10.1002/adma.202418074. 39962841

[83] J. Wang , J. Lin , L. Chen , L. Deng , and W. Cui , “Endogenous Electric‐Field‐Coupled Electrospun Short Fiber via Collecting Wound Exudation,” Advanced Materials 34 (2022): 2108325, 10.1002/adma.202108325. 34902192

[84] M. H. Kim , J. Lee , J. N. Lee , H. Lee , and W. H. Park , “Mussel‐inspired Poly (γ‐glutamic acid)/Nanosilicate Composite Hydrogels With Enhanced Mechanical Properties, Tissue Adhesive Properties, and Skin Tissue Regeneration,” Acta Biomaterialia 123 (2021): 254–262, 10.1016/j.actbio.2021.01.014. 33465509

[85] S.‐S. Jin , D.‐Q. He , D. Luo , et al., “A Biomimetic Hierarchical Nanointerface Orchestrates Macrophage Polarization and Mesenchymal Stem Cell Recruitment to Promote Endogenous Bone Regeneration,” ACS Nano 13 (2019): 6581–6595, 10.1021/acsnano.9b00489. 31125522

[86] T. Deng , D. Gao , X. Song , et al., “A Natural Biological Adhesive From Snail Mucus for Wound Repair,” Nature Communications 14 (2023): 396, 10.1038/s41467-023-35907-4. PMC9873654 36693849

[87] L. J. Draaijers , F. R. H. Tempelman , Y. A. M. Botman , et al., “The Patient and Observer Scar Assessment Scale: A Reliable and Feasible Tool for Scar Evaluation,” Plastic and Reconstructive Surgery 113 (2004): 1960–1965, 10.1097/01.PRS.0000122207.28773.56. 15253184

