# Supplementary material for: Seta‐Inspired Mechano‐Intelligent Janus Bandage with Coordinated Adhesion–Contraction for Minimizing Scarring
Source: Adv Mater. 2026 May 15;38(34):e05122. doi: 10.1002/adma.202505122 (PMC13274697; doi:10.1002/adma.202505122)
Supplement: Supplementary file 1 — Supporting File 1: adma73347‐sup‐0001‐SuppMat.docx. [file ADMA-38-e05122-s002.docx]

Supporting Information

**Seta-inspired mechano-intelligent Janus bandage** **with** **coordinated adhesion-contraction for minimizing scarring**

*Di Suo, Yuhe Yang, Shuai Zhao, Ho-Pan Bei, Avery Chik-Him Lam, Wei-Qiang Tan, Kenneth Kak-yuen Wong, Xin Zhao**

D. Suo, Y. Yang, S. Zhao, H-P. Bei, A. Lam, X. Zhao

Department of Applied Biology and Chemical Technology, The Hong Kong Polytechnic University, Hung Hom, Hong Kong SAR, 999077, PR China

E-mail: xin.zhao@polyu.edu.hk

D. Suo, Y. Yang, S. Zhao, H-P. Bei, X. Zhao

The Hong Kong Polytechnic University Shenzhen Research Institute, Shenzhen, Guangdong 518057, PR China

X. Zhao

Research Institute for Intelligent Wearable Systems, The Hong Kong Polytechnic University, Hung Hom, Kowloon, Hong Kong SAR, 999077, PR China.

W. Tan

Department of Plastic Surgery, Sir Run Run Shaw Hospital, Zhejiang University School of Medicine, 3 East Qingchun Road, Hangzhou, 310016, PR China

K.K. Wong

Department of Surgery, Li Ka Shing Faculty of Medicine, The University of Hong Kong, Pok Fu Lam, Hong Kong SAR, 999077, PR China


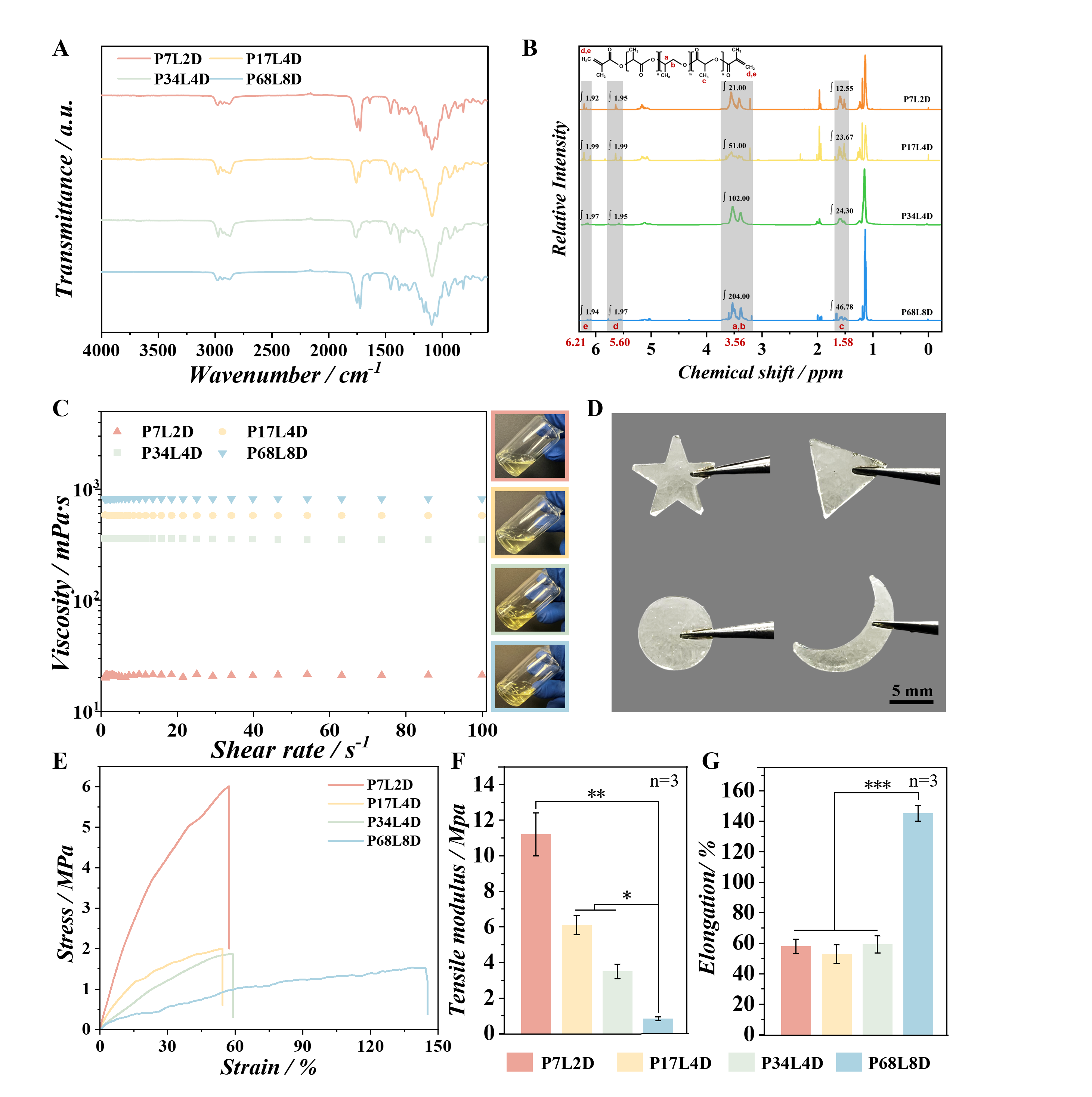


**Figure S1.** Characterization of synthesized poly (lactide-co-propylene glycol-co-lactide) dimethacrylates (PmLnD) (i.e., P7L2D, P17L4D, P34L4D and P68L8D). (A) Fourier transform infrared (FTIR) and (B) ^1^H nuclear magnetic resonance (^1^H NMR) spectra of the PmLnD. (C) Viscosity characterization of the PmLnD. (D) The moldability of P68L8D to different shapes. (E) The tensile stress-strain curve, (F) tensile modulus, and (G) elongation-at-break of the PmLnD. All experiments were conducted with a sample size of *n* = 3 and analyzed using one-way ANOVA followed by Tukey’s post hoc test for multiple comparisons. Data are expressed as mean ± SD, with statistical significance denoted as **p* < 0.05, ***p* < 0.01, and ****p* < 0.001.


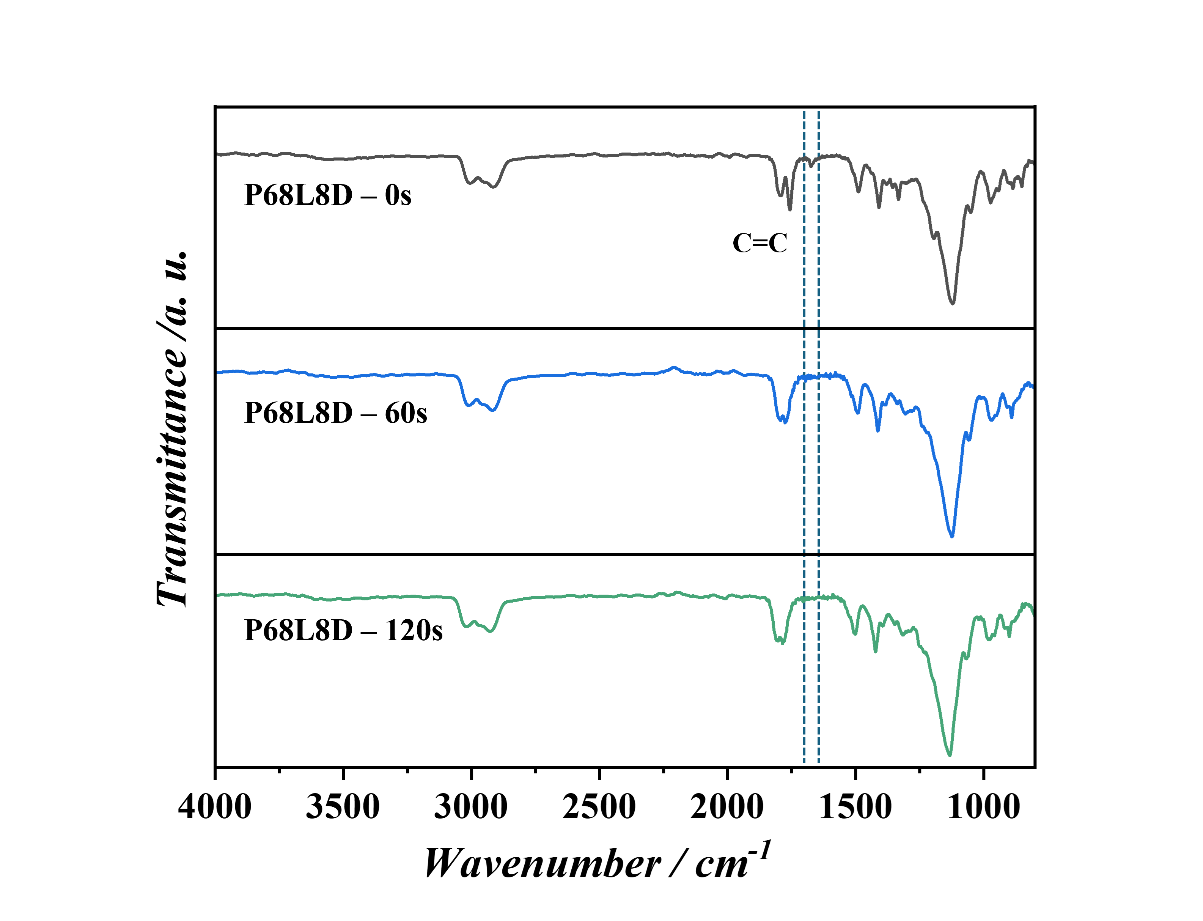


**Figure S2**. FTIR evidence of vinyl-group conversion during photocuring of P68L8D.


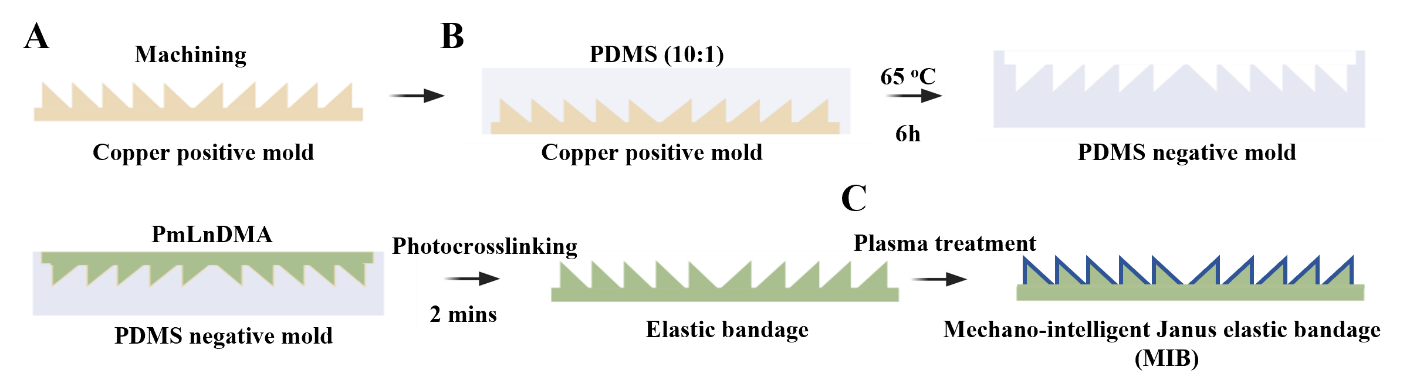


**Figure S3.** Fabrication process of the MIBs. (A) Schematic representation of the template fabrication using precision mechanical machining. (B) Two-step micromolding process for the MIBs. (C) Plasma treatment applied to the interior wedged side of the MIBs**.**

**
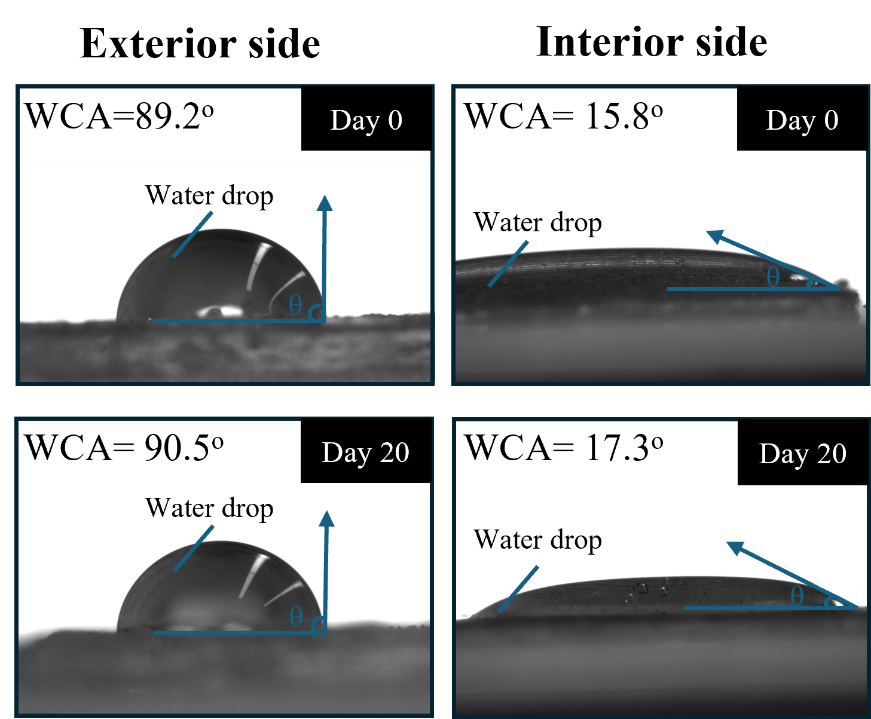
**

**Figure S4**. Contact-angle stability of the plasma-modified surface after sterilization (day 0) and storage (day 20).


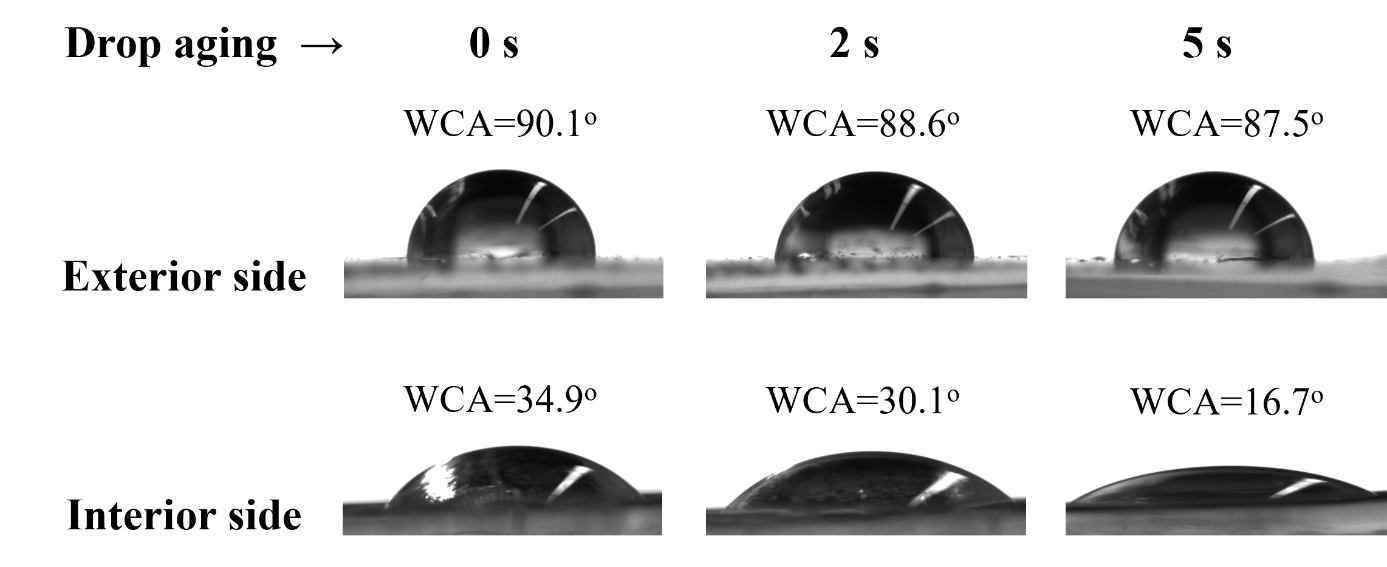


**Figure S5**. Dynamic wetting behavior of the micro-wedged, plasma-treated contacting surface of MIB.

**
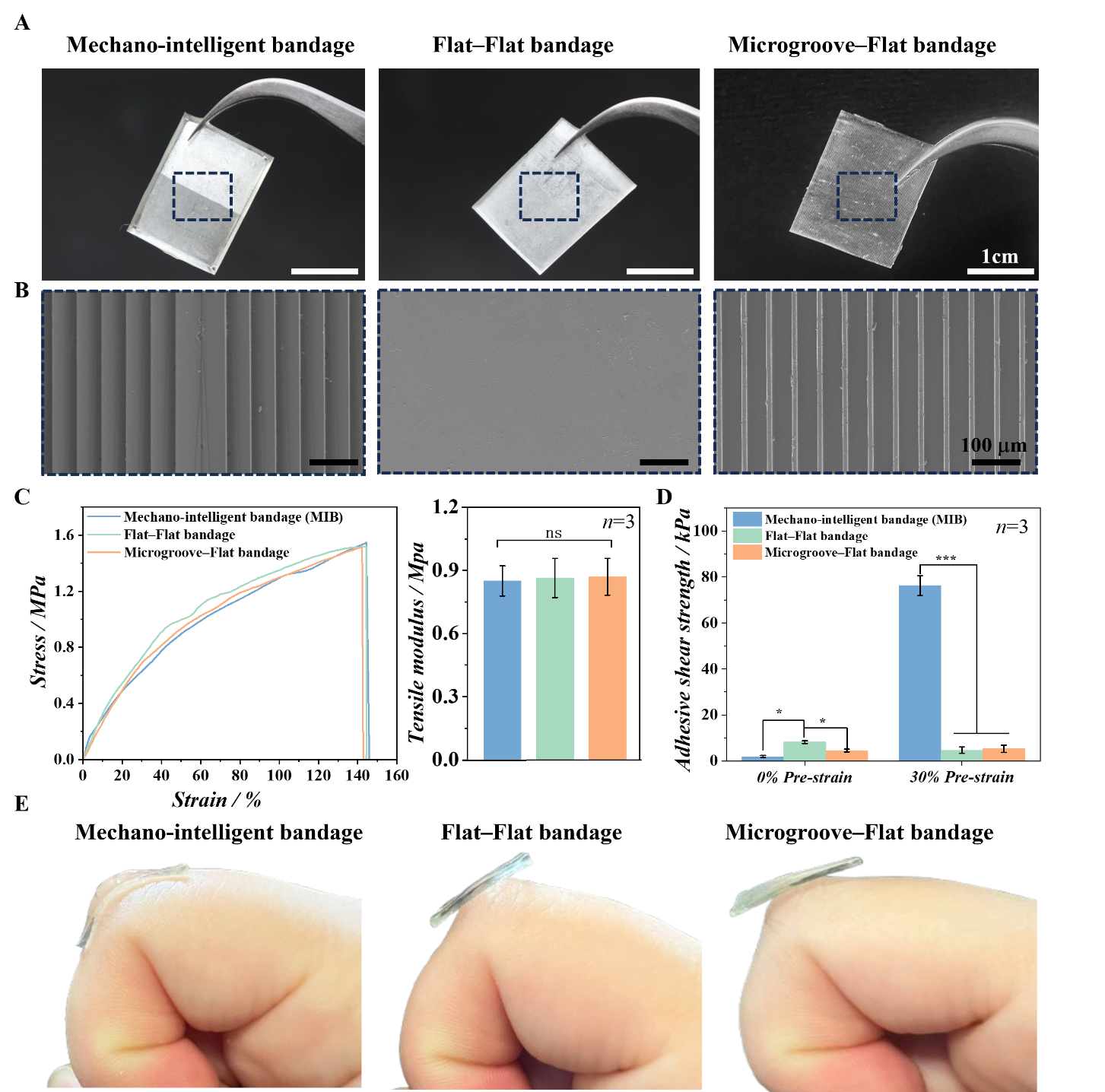
**

**Figure S6**. Design, fabrication, and adhesion performance of different bandage designs. (A) Photographs of the mechano-intelligent bandage (MIB), Flat–Flat bandage, and Microgroove–Flat bandage. (B) SEM images showing the corresponding surface morphologies. (C) Stress–strain curves and comparison of tensile modulus among the three bandages. (D) Quantitative analysis of adhesive shear strength before and after pre-strain treatment. (E) Photographs demonstrating the adhesion of each bandage on finger joint. All experiments were conducted with a sample size of *n* = 3 and analyzed using a one-way or two-way ANOVA followed by Tukey’s post hoc test for multiple comparisons. Data are expressed as mean ± SD, with statistical significance denoted as **p* < 0.05 and ****p* < 0.001.


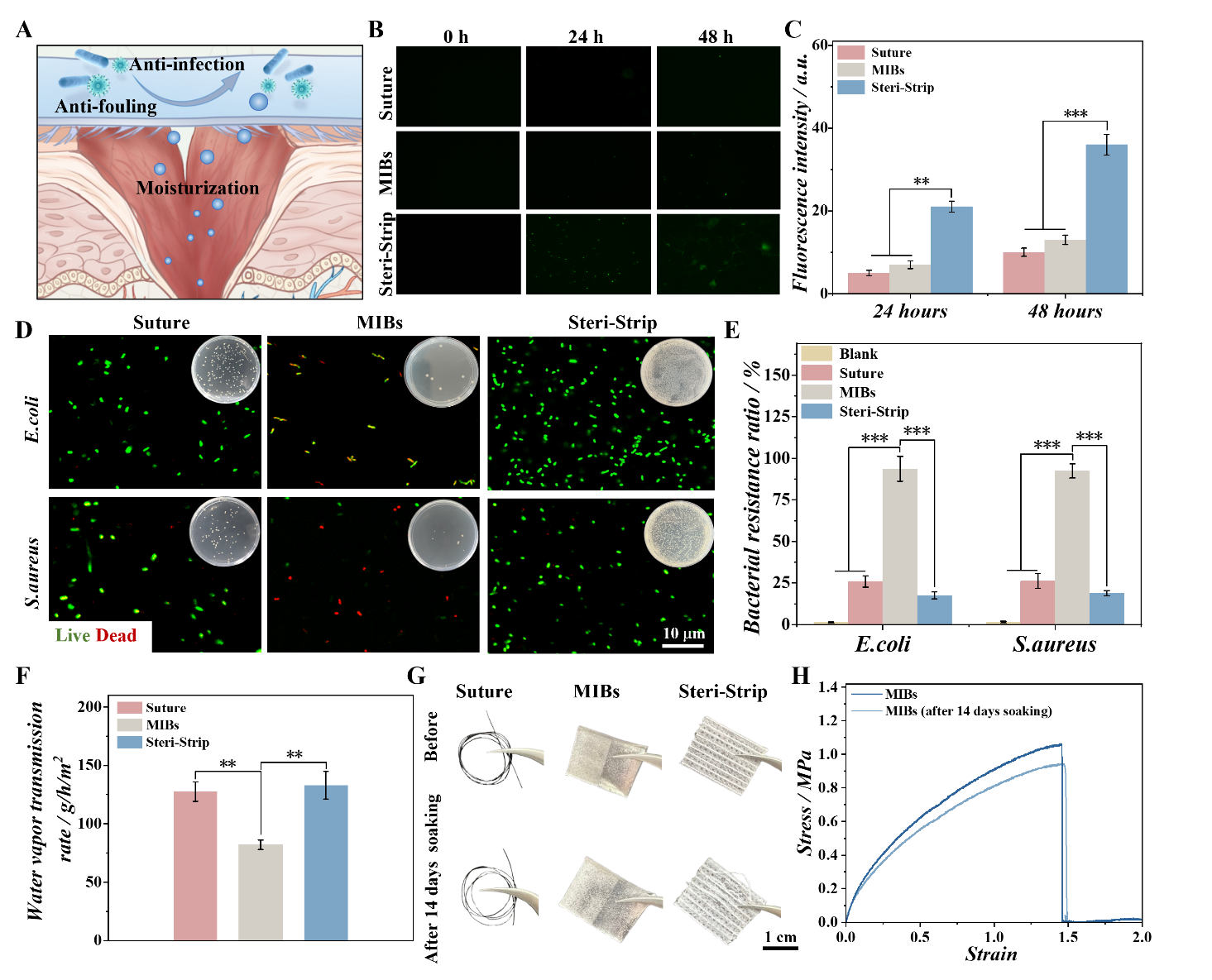


**Figure S7**. Maintenance of a moist and sterile environment with MIBs. (A) Diagram illustrating our MIB for maintaining a moist and sterile environment. (B) Fluorescence images and (C) fluorescence intensity quantification of the adsorbed BSA-FITC protein on different samples. (D) Representative photos of spread plates and fluorescence images after 48 h of incubation of *E. coli* and *S. aureus* with different samples’ exterior surfaces. (E) Quantitative analysis of the *in vitro* antibacterial efficiency. (F) Water vapor transmission rate (WVTR) evaluation of different samples. (G) Representative images of the different samples before and after 14 days of soaking. (H) Comparison of mechanical property of MIBs before and after 14 days soaking. All experiments were conducted with a sample size of *n* = 3 and analyzed using a one-way or two-way ANOVA followed by Tukey’s post hoc test for multiple comparisons. Data are expressed as mean ± SD, with statistical significance denoted as ***p* < 0.01, and ****p* < 0.001.


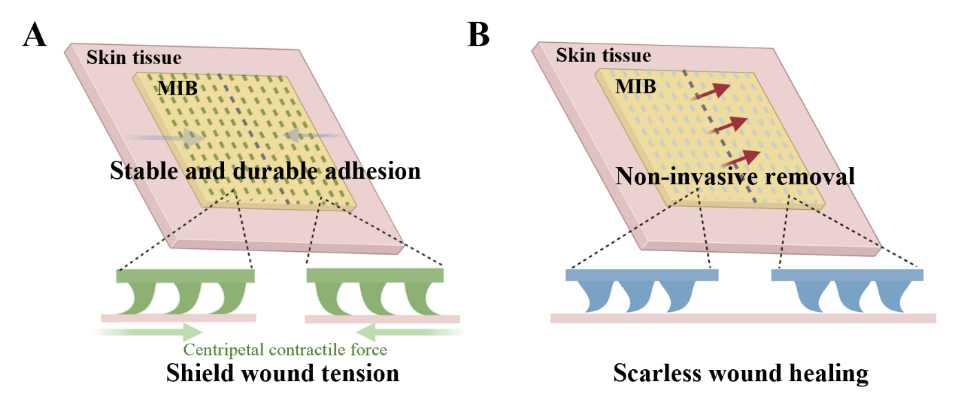


**Figure S8**. Schematic of the mechano-intelligent bandage (MIB) during (A) adhesion for wound closure, tension modulation, and (B) controlled de-adhesion.

**
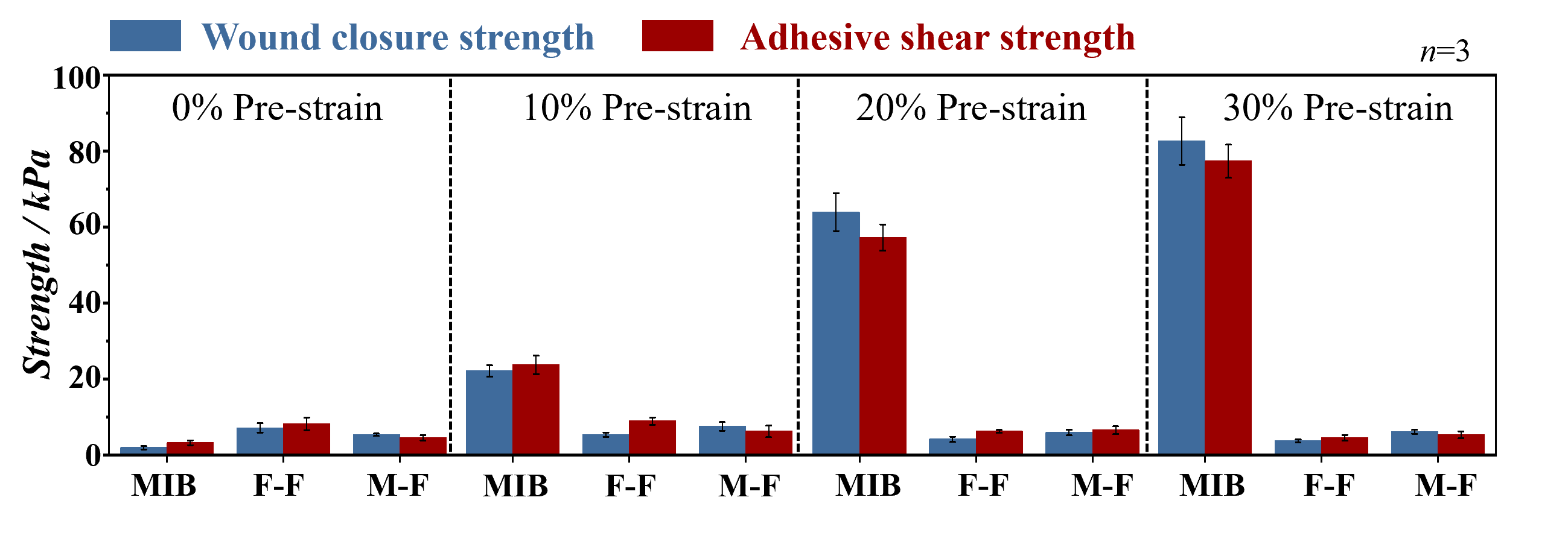
**

**Figure S9**. Evaluation of wound closure strength and adhesion strength for the mechano-intelligent bandage (MIB), Flat-Flat (F-F), and Microgroove-Flat (M-F) bandages under varying pre-strain levels. All experiments were conducted with a sample size of *n* = 3.


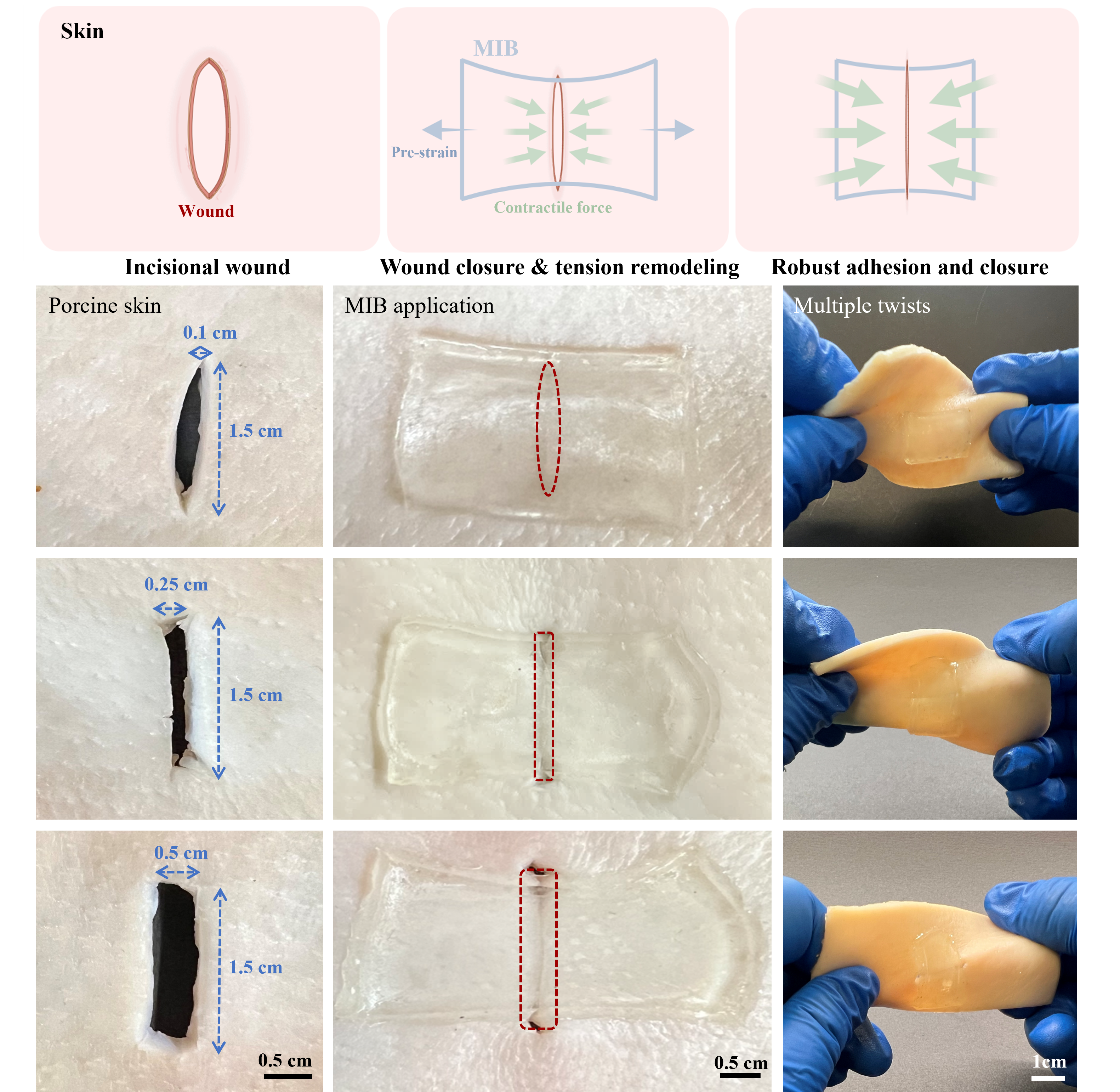


**Figure S10.** *In vitro* wound closure tests with varying wound sizes (0.1 cm× 1.5 cm, 0.25 cm× 1.5 cm, and 0.5 cm× 1.5 cm)


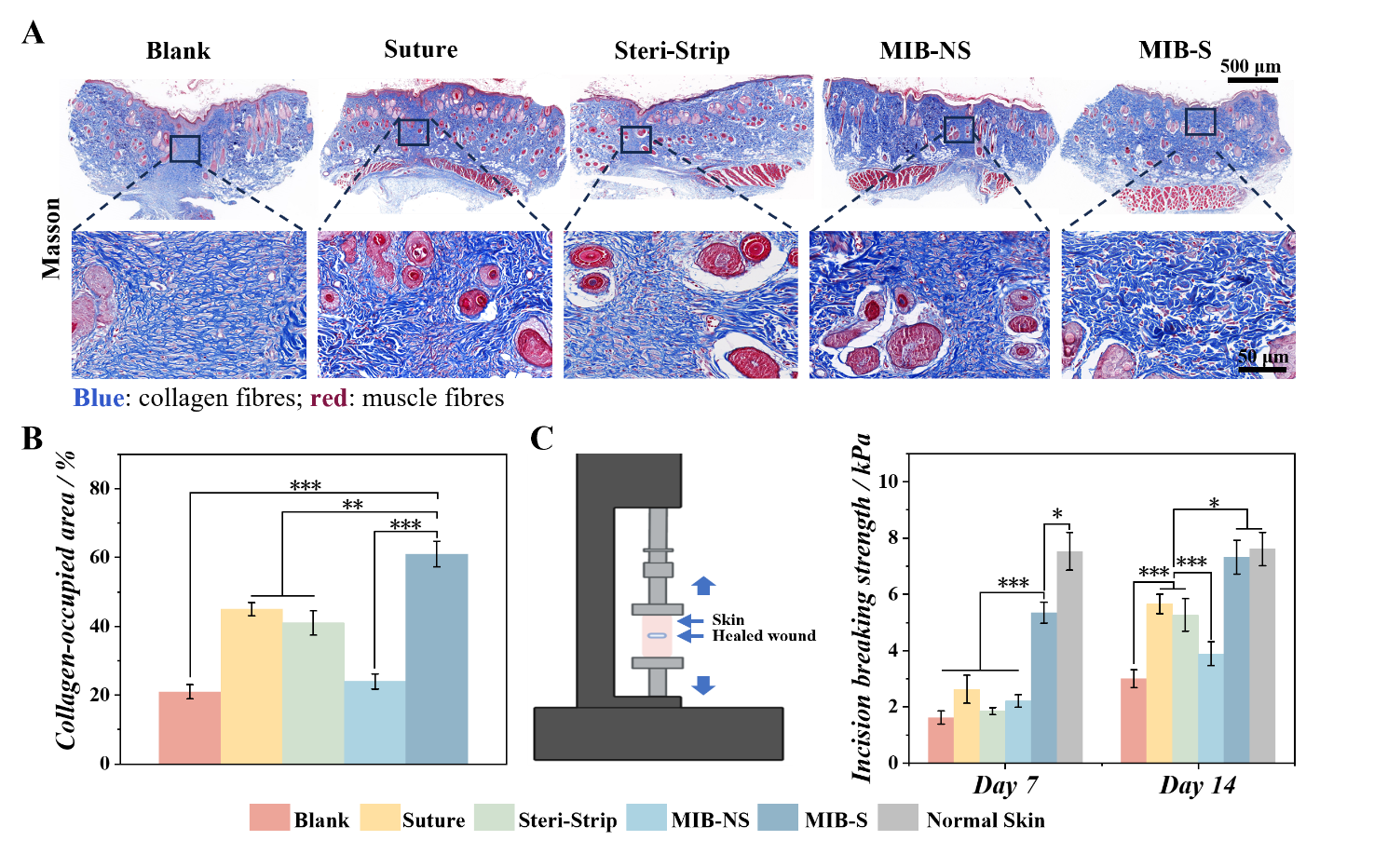


**Figure S11.** (A) Masson’s trichrome staining and (B) the quantification of collagen occupied area for the wound sections after 14 days of treatment. (C) Quantitative analysis of the incision breaking strength of healed wounds after 14 days. All experiments were conducted with a sample size of *n* = 4 and analyzed using a one-way or two-way ANOVA followed by Tukey’s post hoc test for multiple comparisons. Data are expressed as mean ± SD, with statistical significance denoted as **p* < 0.05, ***p* < 0.01, and ****p* < 0.001.


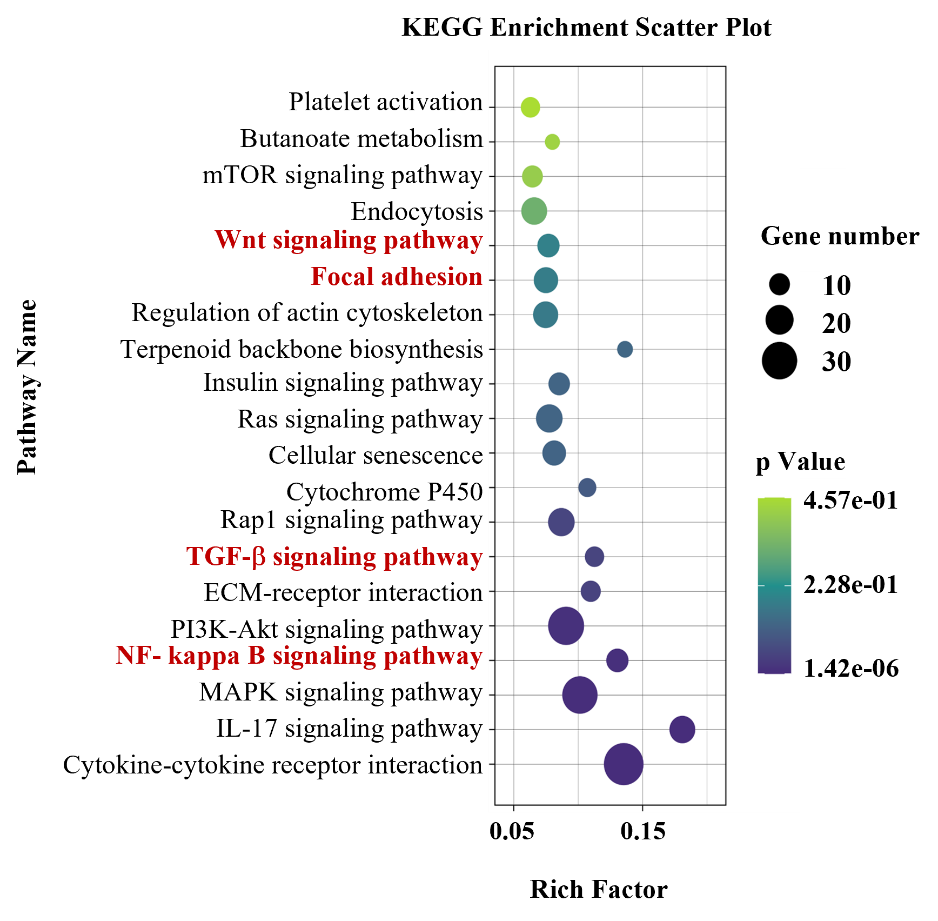


**Figure S12**. KEGG pathways on day 7 (MIB-S group versus blank group).


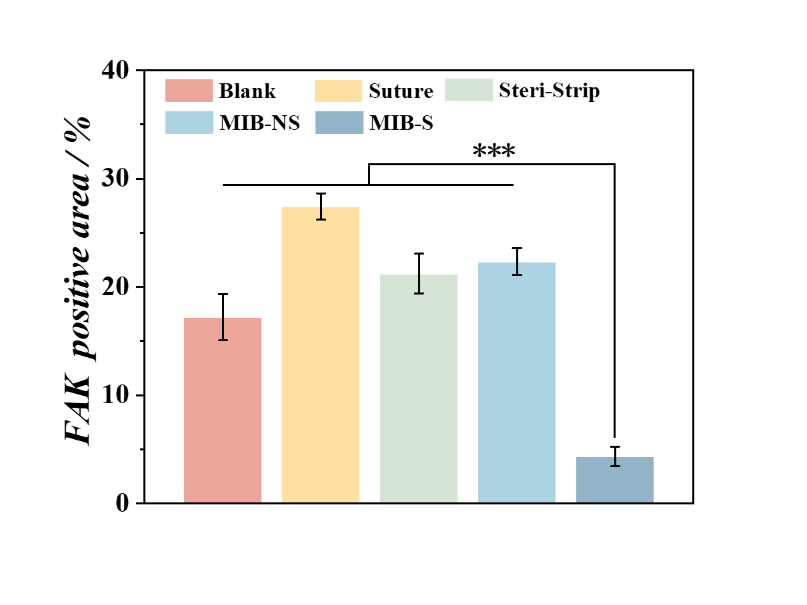


**Fig. S13.** Quantification of immunofluorescence staining of FAK on day 7. All experiments were conducted with a sample size of *n* = 4 and analyzed using one-way ANOVA followed by Tukey’s post hoc test for multiple comparisons. Data are expressed as mean ± SD, with statistical significance denoted as ****p* < 0.001.


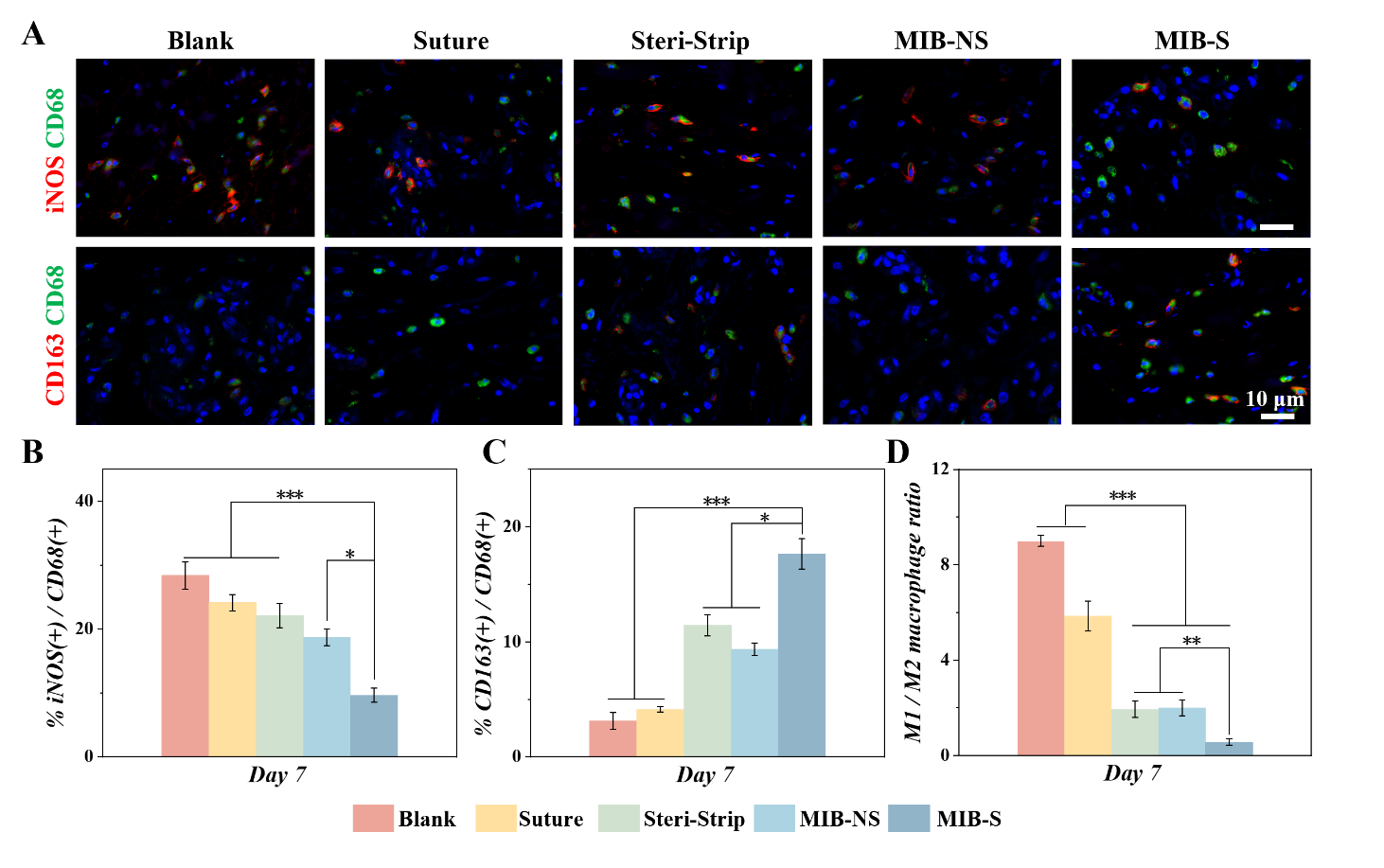


**Figure S14.** (A) Immunofluorescence staining was performed for CD68 (pan-macrophage marker), iNOS (M1 macrophage marker), and CD163 (M2 macrophage marker) on day 7. Quantitative analysis included the proportion of (B) iNOS(+)/CD68(+), (C) CD163(+)/CD68(+), and (D) the M1/M2 macrophage ratio across different treatments on day 7. All experiments were conducted with a sample size of *n* = 4 and analyzed using one-way ANOVA followed by Tukey’s post hoc test for multiple comparisons. Data are expressed as mean ± SD, with statistical significance denoted as **p* < 0.05, ***p* < 0.01, and ****p* < 0.001.


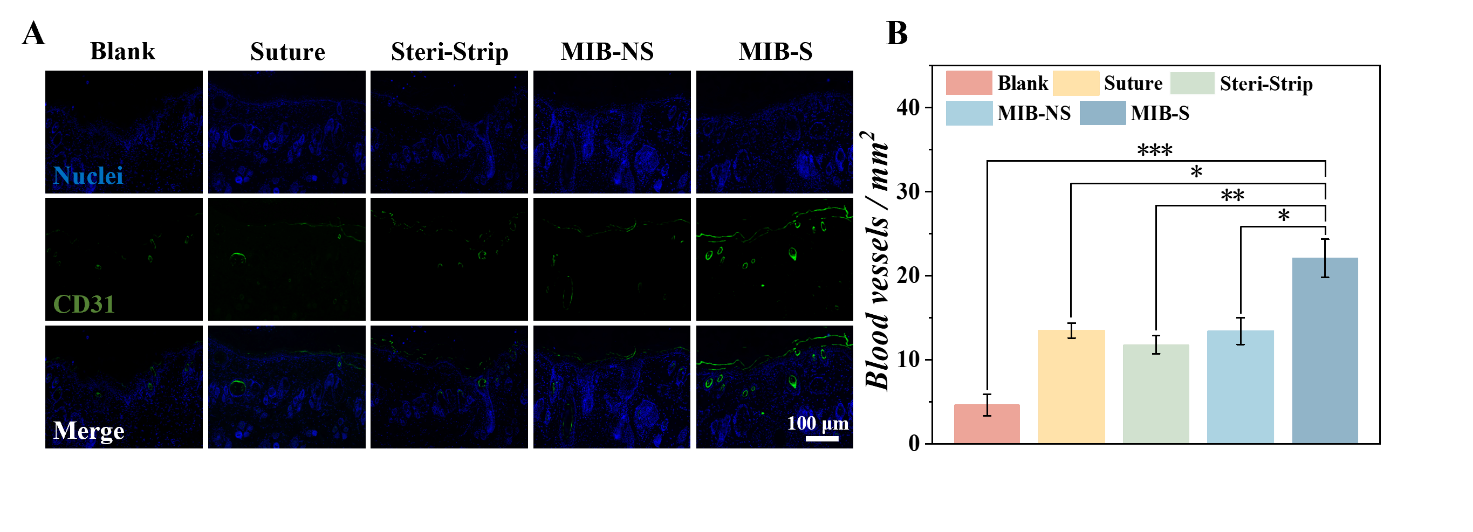


**Figure S15**. (A) Immunofluorescence CD31 staining for the wound after 14 days. The green and blue colour indicated the CD31 and nuclei, respectively. (B) Quantification of the blood vessel density after 14 days. All experiments were conducted with a sample size of *n* = 4 and analyzed using one-way ANOVA followed by Tukey’s post hoc test for multiple comparisons. Data are expressed as mean ± SD, with statistical significance denoted as **p* < 0.05, ***p* < 0.01 and ****p* < 0.001.


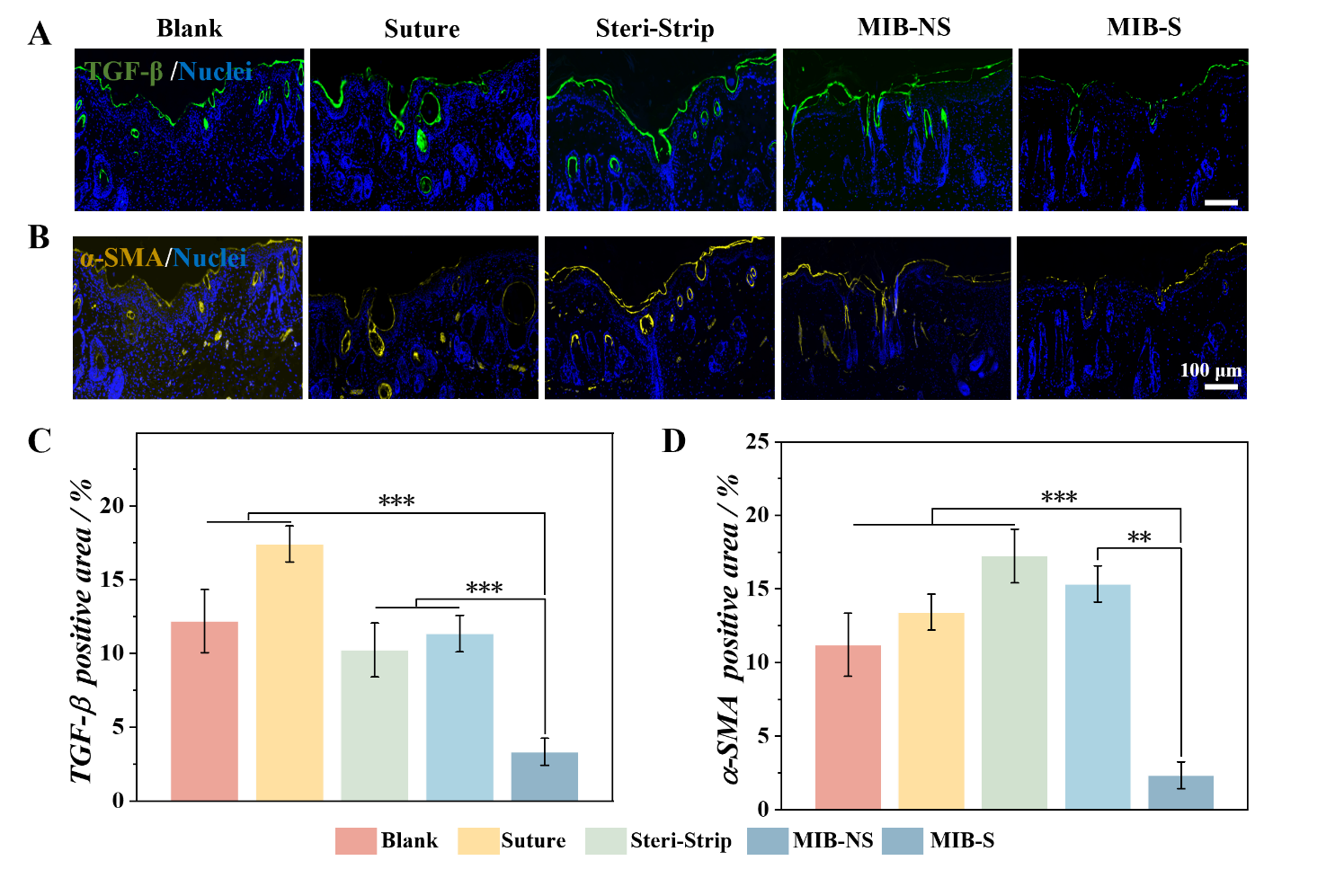


**Figure S16**. Immunofluorescence staining images of (A) Transforming growth factor beta (TGF-β), and (B) α-smooth muscle actin (α-SMA) positive cells in wounds treated with different groups on day 14. Cell nuclei were stained with DAPI (blue). Quantification of immunofluorescence staining of (C) TGF-β, and (D) α-SMA on day 14. Sample size *n* = 4 for all experiments by a one-way ANOVA with a Tukey’s post hoc test for multiple comparisons. Data are presented as mean ± SD. ***p* < 0.01 and ****p* < 0.001 are considered the statistical significance.


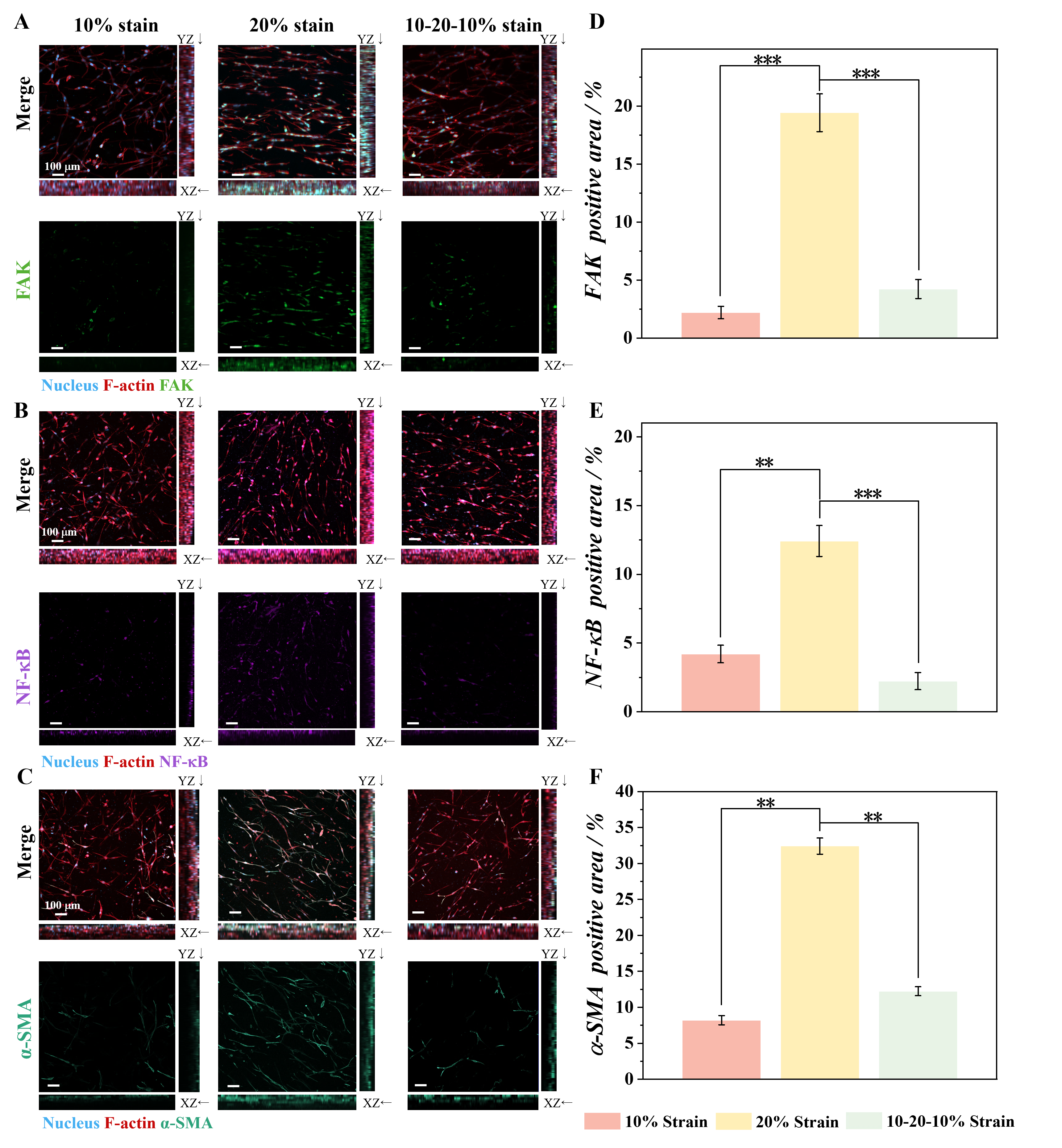


**Figure S17**. Representative confocal z-stack images of (A) FAK, (B) NF-κB, and (C) α-SMA in NIH 3T3 fibroblasts embedded in a type I collagen matrix under 10% constant strain (physiological skin tension), 20% constant strain (elevated wound tension), and a stepwise tension-unloading trajectory (10-20-10% strain) mimicking MIB-mediated tension release. Positive area (%) quantification of (D) FAK, (E) NF-κB, and (F) α-SMA in 10%, 20%, and 10-20-10% strain groups, respectively. Sample size *n =* 3 for all experiments by a one-way ANOVA with a Tukey’s post hoc test for multiple comparisons. Data are presented as mean ± SD. ***p* < 0.01 and ****p* < 0.001 are considered the statistical significance.


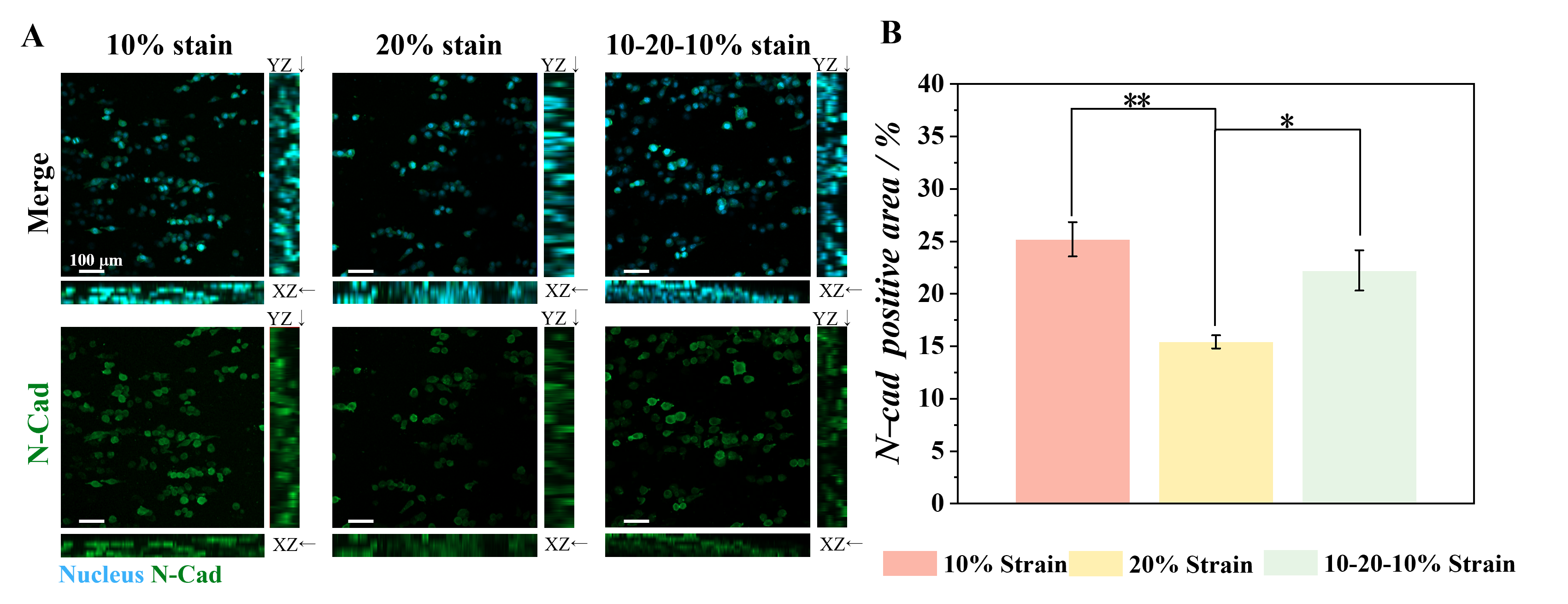


**Figure S18**. (A) Representative confocal z-stack images and (B) positive area (%) quantification of N-cadherin (N-Cad) in HaCaT keratinocytes embedded in a type I collagen matrix under 10% constant strain, 20% constant strain, or tension-unloading trajectory (10-20-10% strain). Sample size *n =* 3 for all experiments by a one-way ANOVA with a Tukey’s post hoc test for multiple comparisons. Data are presented as mean ± SD. **p* < 0.05 and ***p* < 0.01 are considered the statistical significance.

**Table S1.** Comparison between the bioinspired mechano-intelligent Janus bandage (MIB) with prior gecko-inspired bandages (GIBs)

| GIBs | Structural design | Contractile force programming | Adhesion-contraction coupling | Reversible detachment | Ref |
| --- | --- | --- | --- | --- | --- |
| 1 | Unidirectional | × | × | √ | ^[1]^ |
| 2 | Unidirectional | × | × | √ | ^[2]^ |
| 3 | Unidirectional | √ | × | × | ^[3]^ |
| 4 | Unidirectional | √ | × | × | ^[4]^ |
| 5 | Unidirectional | × | × | × | ^[5]^ |
| 6 | Unidirectional | × | × | × | ^[6]^ |
| 7 | Unidirectional | × | × | √ | ^[7]^ |
| 8 | Unidirectional | × | × | √ | ^[8]^ |
| 9 | Unidirectional | × | × | √ | ^[9]^ |
| 10 | Unidirectional | × | × | × | ^[10]^ |
| **MIB** | **Bidirectional** | **√** | **√** | **√** |  |

**Table S2.** Quantification of PPG and LA segment lengths and methacrylation efficiency of PmLnD based on ^1^H NMR peak integration.

| **Sample** | **∫ _3.56_ (PPG, a,b)** | **m = ∫ _3.56_ /3** | **∫ _1.58_ (LA-CH_3_, c)** | **n = ∫ _1.58 /_ 6** | **∫ _6.21_ (vinyl, e)** | **∫ _5.60_ (vinyl, d)** | **DM (%)** |
| --- | --- | --- | --- | --- | --- | --- | --- |
| P7L2D | 21.00 | 7.00 | 12.55 | 2.09 | 1.92 | 1.95 | 96.75 |
| P17L4D | 51.00 | 17.00 | 23.67 | 3.95 | 1.99 | 1.99 | 99.50 |
| P34L4D | 102.00 | 34.00 | 24.30 | 4.05 | 1.97 | 1.95 | 98.00 |
| P68L8D | 204.00 | 68.00 | 46.78 | 7.80 | 1.94 | 1.97 | 97.75 |

*Integrations were normalized to the PPG backbone resonance at 3.56 ppm

**Table S3.** Primers sequences for RT-QPCR

| Primer name | Sequence (5' to 3') |
| --- | --- |
| Cxcl2-F | CATCCAGAGCTTGAGTGTGACG |
| Cxcl2-R | GGCTTCAGGGTCAAGGCAAACT |
| Ptgs2-F | CTTGAACACGGACTTGCTCAC |
| Ptgs2-R | GCCTTTGCCACTGCTTGTA |
| IL1B-F | TGGACCTTCCAGGATGAGGACA |
| IL1B-R | GTTCATCTCGGAGCCTGTAGTG |
| Fgf18-F | CACTTTCTACTGCTGTGCTTCCA |
| Fgf18-R | GCATACTTGTCCCCGTCCTC |
| Krt8-F | ACAAGGTAGAGCTGGAGTCTCG |
| Krt8-R | AGCACCACAGATGTGTCCGAGA |
| wNT7A-F | TTCGCCAAGGTCTTCGTGGATG |
| wNT7A-R | TACAGGAGCCTGACACACCATG |
| mmp3-f | CTCTGGAACCTGAGACATCACC |
| mmp3-r | AGGAGTCCTGAGAGATTTGCGC |
| JUN-F | CGTTAACAGTGGGTGCCAACTC |
| JUN-R | TCGCAACCAGTCAAGTTCTCAAG |
| CCN1-F | GTGAAGTGCGTCCTTGTGGACA |
| CCN1-R | CTTGACACTGGAGCATCCTGCA |
| GAPDH-F | CATCACTGCCACCCAGAAGACTG |
| GAPDH-R | ATGCCAGTGAGCTTCCCGTTCAG |

**Instructions for Use (IFU) of MIB**

**Purpose:**

This IFU provides a step-by-step guide for clinicians to apply the MIB with a target pre-strain (e.g., 30% pre-strain) to ensure optimal adhesion-contraction coordination and scarless wound healing.

**Step-by-Step Application Instructions:**

(1) Preparation:

- Clean and dry the wound area thoroughly.
- Ensure the wound edges are aligned appropriately for closure.
- Remove the MIB from sterile packaging and inspect for damage or contamination.

(2) Measuring Pre-Strain:

- Identify the unstretched length of the MIB (denoted as L0).
- Stretch the MIB to 1.3 times its original length (e.g., 30% pre-strain), ensuring the elongated length is L = 1.3 × L0.
- Use the included pre-strain ruler or stretch guide to confirm the correct elongation visually.

(3) Application:

- While maintaining the pre-strain, position the MIB over the wound area.
- Secure one end of the bandage to the skin gently using light pressure.
- Gradually release the stretched section as you adhere the bandage along the wound’s length, ensuring even tension distribution.

(4) Final Check:

- Confirm that the bandage is securely attached and aligned with the wound edges.
- Verify that the MIB is under consistent tension across its length.

**Estimated Time to Apply:**

- Initial Setup (Untrained Clinician): ~5-7 minutes.
- Post-Learning Phase (Trained Clinician): ~2-3 minutes.

**Common User Errors:**

(1) Insufficient Pre-Strain:

- Clinicians may fail to stretch the MIB to the target length, resulting in suboptimal contractile force.
- Solution: Use the pre-strain ruler or markings on the bandage for visual guidance.

(2) Excessive Pre-Strain:

- Over-stretching the MIB beyond the recommended 30% can cause tissue ischemia or discomfort.
- Solution: Adhere to the pre-strain guidelines and verify the elongation using the ruler.

(3) Uneven Application:

- Misalignment of the bandage can lead to inconsistent tension across the wound.
- Solution: Apply the bandage slowly, ensuring even pressure along both edges of the wound.

(4) Premature Detachment:

- Early removal of the MIB can disrupt the healing process.
- Solution: Follow the recommended removal timeline (e.g., after 7-14 days or as instructed).

**Learning Curve Observations:**

(1) Initial Training:

- Clinicians may require 2-3 supervised applications to become proficient in achieving the target pre-strain and applying the MIB correctly.

(2) Improved Efficiency:

- After the initial learning phase, the application time reduces significantly, with clinicians achieving consistent pre-strain and alignment within 2-3 minutes.

(3) Feedback from Users:

- Clinicians report that the pre-strain ruler and guided instructions are highly effective in minimizing errors during the learning phase.


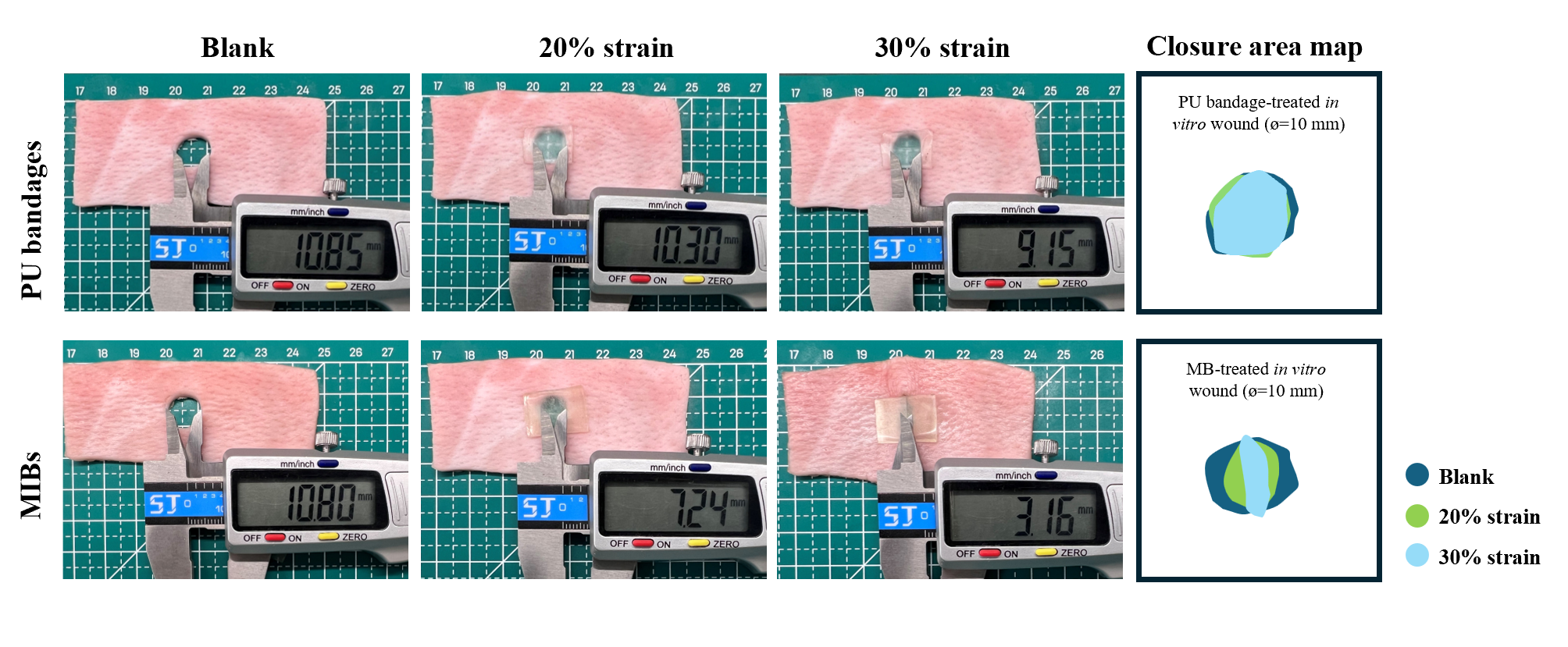


**Figure S19.** Circular-opening closure test on porcine skin. Representative photographs showing the characteristic diameter of a circular opening measured by a digital caliper before and after applying the bandage constructs.

References

1. J. Yu, S. Chary, S. Das, J. Tamelier, et al., Gecko-inspired dry adhesive for robotic applications, *Adv. Funct. Mater.* **2011**, *21*, 3010.

<https://doi.org/10.1002/adfm.201100493>

2. S. Chary, J. Tamelier, K. Turner, A microfabricated gecko-inspired controllable and reusable dry adhesive, *Smart Mater. Struct.* **2013**, *22*, 025013.

<https://doi.org/10.1088/0964-1726/22/2/025013>

3. Y. Tian, N. Pesika, H. Zeng, et al., Adhesion and friction in gecko toe attachment and detachment, *Proc. Natl. Acad. Sci. U. S. A* **2006**, *103*, 19320.

<https://doi.org/10.1073/pnas.0608841103>

4. H. Lee, B. P. Lee, P. B. Messersmith, A reversible wet/dry adhesive inspired by mussels and geckos, *Nature* **2007**, *448*, 338.

<https://doi.org/10.1038/nature05968>

5. B. Aksak, M. P. Murphy, M. Sitti, Adhesion of biologically inspired vertical and angled polymer microfiber arrays, *Langmuir* **2007**, *23*, 3322.

<https://doi.org/10.1021/la062697t>

6. M. P. Murphy, B. Aksak, M. Sitti, Gecko-inspired directional and controllable adhesion**,** *Small* **2009**, *5*, 170.

<https://doi.org/10.1002/smll.200801161>

7. L. F. Boesel, C. Greiner, E. Arzt, A. Del Campo, Gecko-inspired surfaces: a path to strong and reversible dry adhesives, *Adv. Mater.* **2010**, *22*, 2125.

<https://doi.org/10.1002/adma.200903200>

8. Y. Ma, S. Ma, Y. Wu, et al., Remote control over underwater dynamic attachment/detachment and locomotion, *Adv. Mater.* **2018**, *30*, 1801595.

<https://doi.org/10.1002/adma.201801595>

9. H. Shahsavan, S. M. Salili, A. Jákli, B. Zhao, Thermally active liquid crystal network gripper mimicking the self-peeling of gecko toe pads, *Adv. Mater.* **2017**, *29*, 1604021.

<https://doi.org/10.1002/adma.201604021>

10. S. Lee, W. Kim, H. Sharma, M. Safdar, D. Kim, C. Park, J. Kim, Ultratiny scale patterned biomedical janus patch with adhesive and anti-adhesive properties, *Nano Lett.* **2025**, *25*, 12470.

<https://doi.org/10.1021/acs.nanolett.5c02286>
